# Vaccine genetics of IGHV1-2 VRC01-class broadly neutralizing antibody precursor naïve human B cells

**DOI:** 10.1038/s41541-021-00376-7

**Published:** 2021-09-06

**Authors:** Jeong Hyun Lee, Laura Toy, Justin T. Kos, Yana Safonova, William R. Schief, Colin Havenar-Daughton, Corey T. Watson, Shane Crotty

**Affiliations:** 1 Center for Infectious Disease and Vaccine Research, La Jolla Institute for Immunology (LJI), La Jolla, CA USA; 2 Consortium for HIV/AIDS Vaccine Development (CHAVD), The Scripps Research Institute, La Jolla, CA USA; 3 Department of Biochemistry and Molecular Genetics, University of Louisville School of Medicine, Louisville, KY USA; 4 Computer Science and Engineering Department, University of California San Diego, San Diego, CA USA; 5 International AIDS Vaccine Initiative Neutralizing Antibody Center, The Scripps Research Institute, La Jolla, CA USA; 6 Department of Immunology and Microbiology, The Scripps Research Institute, La Jolla, CA USA; 7 Ragon Institute of Massachusetts General Hospital, Massachusetts Institute of Technology and Harvard University, Cambridge, MA USA; 8 Department of Medicine, Division of Infectious Diseases and Global Public Health, University of California, San Diego (UCSD), La Jolla, CA USA

**Keywords:** Protein vaccines, Humoral immunity

## Abstract

A successful HIV vaccine eliciting broadly neutralizing antibodies (bnAbs) must overcome the hurdle of being able to activate naive precursor B cells encoding features within their germline B cell receptors (BCR) that allow recognition of broadly neutralizing epitopes. Knowledge of whether bnAb precursor B cells are circulating at sufficient frequencies within individuals in communities heavily impacted by HIV may be important. Using a germline-targeting eOD-GT8 immunogen and high-throughput droplet-based single-cell BCR sequencing, we demonstrate that large numbers of paired BCR sequences from multiple donors can be efficiently screened to elucidate precursor frequencies of rare, naive VRC01-class B cells. Further, we analyzed IGHV1-2 allelic usage among three different cohorts; we find that IGHV1-2 alleles traditionally thought to be incompatible with VRC01-class responses are relatively common in various human populations and that germline variation within IGHV1-2 associates with gene usage frequencies in the naive BCR repertoire.

## Introduction

Broadly neutralizing antibodies (bnAbs) are present in a minority of patients chronically infected with human immunodeficiency virus-1 (HIV)^1–3^. These antibodies achieve neutralization breadth and potency against diverse circulating clinical strains by accruing high numbers of somatic hypermutations (SHMs), allowing B cells to efficiently bind to conserved epitopes on the HIV Envelope viral spike protein (Env). BnAb structural and genetic analyses have shown that many bnAb features required for broad and potent neutralization, such as specific CDR lengths^4–7^ and certain amino acid residues at fixed positions defined by immunoglobulin (IG) variable (V), diversity (D), or joining (J) gene usage^8,9^, are predetermined by recombined naive B cell receptors (BCRs). The majority of B cells in the human repertoire do not have BCRs with the potential to become HIV bnAbs. Thus, vaccine priming of rare bnAb precursor B cells likely requires custom immunogens designed to bind specifically to targeted precursors^10^. Making the problem even more challenging, inferred germline (iGL) precursors for many potent HIV bnAbs have been found to have very low or no detectable affinity for wild-type HIV Env^11–17^, and wild-type Env immunogens have not succeeded in eliciting bnAb responses^18^. This lack of affinity of bnAb precursors for wild-type HIV Env remains one of the main impediments in neutralizing antibody-directed HIV vaccine efforts.

One theoretical approach to recapitulating bnAb responses via vaccination involves priming with an immunogen that has exceptionally high affinity for bnAb precursors, then sequentially introducing more native Env-like immunogens to drive bnAb class SHMs^19^. Such priming immunogens are fittingly described as germline targeting (GT) priming immunogens^20^, and a sequential vaccination strategy anchored by these priming immunogens has been described as “germline-targeting vaccine design”^21,22^. Several GT priming immunogens have been designed specifically to bind the iGL versions of known bnAbs with high affinity^15,16,21–26^. For GT priming immunogens to be efficacious, at least two biological prerequisites must be met; the majority of the human population must have the genetic capacity to encode the targeted germline B cells^16,23,27,28^, and the frequency of such B cells needs to be high enough that they can respond to the immunogen while simultaneously competing against off-target B cells during affinity maturation^16,18,22,23,29–31^.

Using carefully controlled mouse models, it has been shown that parameters that can be used to predict how well an immunogen will perform include: the target B cell precursor frequency, the monovalent affinity of the precursor B cell to the immunogen, and the avidity/multivalency of the immunogen^27,29,32–35^. Because the starting precursor frequency of target B cells in humans cannot be manipulated, it is a key parameter according to which immunogens need to be iteratively designed in order to increase the target affinity. We have previously developed a strategy to directly quantify bnAb B cell precursor frequencies from the human B cell repertoire, by using high-affinity GT probes to isolate antigen-specific naive B cells from the blood of healthy individuals^22,23,36^. One class of bnAbs that were analyzed by this method was precursors to Env CD4-binding site (CD4bs) targeting bnAbs, termed VRC01-class^37^. VRC01-class BCRs are identifiable by the use of the immunoglobulin (IG) heavy chain (HC) variable gene (IGHV) IGHV1-2 paired with a light chain (LC) with a short complementarity determining region 3 (CDR3) of 5 amino acids (5-AA)^38–40^. The engineered outer domain (eOD)-derived GT immunogen eOD-GT8 is designed to bind precursors of VRC01-class B cells. eOD-GT8 was able to bind VRC01-class precursor naive B cells in human blood samples^23,36^, and its derivative, eOD-GT5 60mer, was able to activate germline naive VRC01-class B cells at 1 in 1 million precursor frequency in a small animal model^29^. These were among the key findings that helped advance eOD-GT8-60mer to phase 1 clinical trial as a GT priming immunogen HIV vaccine candidate (NCT03547245).

In previous human B cell repertoire screening for antigen-specific naive B cells, antigen-probe-binding B cells were single cell sorted and then subjected to nested polymerase chain reactions (PCR) performed separately for the BCR HC and LC kappa (IGK) and lambda (IGL) genes. While this method can be efficient when querying a small number of B cells, it becomes overly time consuming and costly as the number of analyzed BCR sequences increases. With improved droplet single-cell RNA sequencing (scRNA-seq) technologies, it is possible to efficiently recover single-cell transcriptomic data from bulk sorted cells. Recently, several groups have performed high-throughput antigen-specific B cell repertoire sequencing using the single-cell immune profiling platform from 10× Genomics^41–44^. However, no studies to our knowledge have used this technology to specifically sort rare antigen-specific naive human B cells. Here we analyzed peripheral B cells from nine healthy donors (Table 1) using droplet-based scRNA-seq to obtain HC and LC paired VRC01-class naive human BCR sequences and demonstrated that this method can reliably identify rare antigen-specific B cells. Additionally, we used these data along with other samples from ethnically diverse population cohorts to analyze the human population genetics of our VRC01-class bnAb-targeting vaccine.

**Table 1:** Summary of all donors from whom BCR sequences were analyzed.

Donor	Probe used	Sequencing method	Data shown
1	Tetramer	10× Genomics	Fig. 1
2	Tetramer	10× Genomics	Fig. 1
3	Tetramer	10× Genomics	Fig. 1
4	60mer	10× Genomics	Fig. 2
5	60mer^a^	10× Genomics	Fig. 2
6	60mer^a^	10× Genomics	Fig. 2
7	60mer^b^	10× Genomics	Fig. 2
8	60mer	Sanger	Supplementary Fig. 2
9	Tetramer and 60mer	10× Genomics	Fig. 3

^a^PBMCs were AlexaFluor647-enriched by first adding AlexaFluor647-conjugated eOD-GT8-60mer, then stained with AlexaFluor488: eOD-GT8-60mer and Pacific-Blue: eOD-GT8^KO^-60mer post-enrichment.

^b^In donor 7, the eOD-GT8^KO^-60mer probe was allowed to bind B cells first, followed by the addition of eOD-GT8-60mers. For samples other than donors 5–7, all eOD-GT8 and eOD-GT8^KO^ probes were added simultaneously.

## Results

### Identification of naive VRC01-class B cells using tetramer probes and high-throughput sequencing

Several CD4bs GT immunogens have been designed to bind VRC01-class precursor BCRs, some of which use the engineered outer domain of gp120 as the base molecule^16,23^. Of these, eOD-GT8 has an average of ~6 nM affinity to several inferred germline (iGL) versions of VRC01-class bnAbs^23^. By tethering biotinylated eOD-GT8 monomers to fluorescently labeled streptavidin (SA) to generate tetramers, we previously isolated CD4bs-specific B cells from the human naive B cell repertoire and identified eOD-GT8-binding VRC01-class naive B cells by single-cell BCR sequencing to reveal that these cells are found in healthy humans at a frequency of ~1 in 300,000 naive B cells^23,36^.

**Fig. 1:**
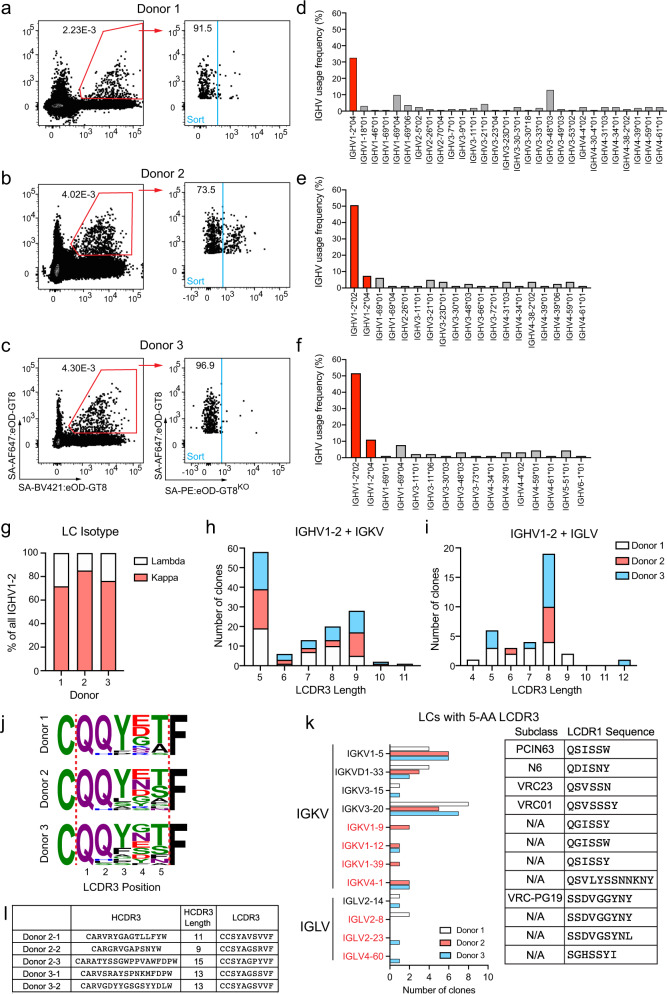
eOD-GT8 tetramer sorted VRC01-class naive B cells. B cells enriched from healthy donor PBMCs were stained with SA: eOD-GT8 tetramers, and sorted for IgG^neg^/eOD-GT8^++^eOD-GT8^KOneg^ B cells, followed by BCR sequencing. **a**–**c** Flow cytometry of sorted cells in donor 1 (**a**), donor 2 (**b**), and donor 3 (**c**). Sorted cells are from the gate shown in blue. **d**–**f** IGHV gene usage distribution of paired IgG^neg^/eOD-GT8^++^eOD-GT8^KOneg^ B cells from donor 1 (**d**), donor 2 (**e**), and donor 3 (**f**). Bars corresponding to IGHV1-2 genes are colored in red. **g** Isotype distribution of LCs coexpressed with IGHV1-2. **h** LCDR3 lengths of IGKV LCs coexpressed with IGHV1-2. **i** As in **h**, but for IGLV LCs. **j** LCDR3 sequences of naive 5-AA IGKV LCs. **k** IGKV and IGLV usage distribution among eOD-GT8^++^eOD-GT8^KOneg^ 5-AA LCDR3 VRC01-class naive B cells and their germline encoded LCDR1 sequences (right). IGKV and IGLV genes shown in black are genes observed in known VRC01-class bnAbs, whereas those indicated in red have not been observed. **l** IOMA-class B cells identified using tetramer probes.

To determine whether droplet-based scRNA-seq was applicable to sequencing rare antigen-specific B cells, we sorted eOD-GT8-specific naive B cells from peripheral blood mononuclear cells (PBMCs) of three independent healthy donors and used the 10× Genomics Chromium platform to obtain BCR sequences (Fig. 1 and Supplementary Fig. 1). As in our previous experiments, antigen-specific cells were defined as those that bound SA: eOD-GT8 on two different fluorescent colors (eOD-GT8^++^) while not binding the eOD-GT8 knockout-II (eOD-GT8^KO^) probe (Fig. 1a–c), which is identical to eOD-GT8 except for mutations in the CD4bs that prevents VRC01-class B cells and their respective iGL BCRs from binding^32,36^.

We first filtered the annotated VDJ sequences. Cell barcodes associated with doublets were identified by the presence of two HC contigs and/or two LC contigs of the same isotype (e.g., two IGLV or two IGKV contigs). Doublets and cells without a HC-LC pair were eliminated from analysis. Because the primers in this system were designed to engage all IGH constant region genes, we were able to identify any IgA^+^ or IgG-subclass^+^ BCRs and removed them from analysis as well. Lastly, cells sorted from donors 2 and 3 were multiplexed with other sort samples by hashtag feature barcoding^45^, and cell barcodes associated with high dual hashtag counts were removed. The final numbers of recovered paired BCR sequences were 163, 81, and 114 in donors 1–3, respectively (Table 2). The IGHV1-2 gene was highly enriched among paired BCR sequences. Across the three donors, between 32 and 60% of the HCs were IGHV1-2 (Fig. 1d–f). In all, 71–86% of LC paired with IGHV1-2 were IGKV (Fig. 1g). Also, 37–43% of the LCs paired with a IGHV1-2 HC had a short 5-AA LCDR3 (42–50% IGKV and 0–21% IGLV, Fig. 1h, i). The frequencies of tetramer-identified eOD-GT8^++^eOD-GT8^KOneg^ IGHV1-2 HCs and the proportion of 5-AA LCDR3 LCs among IGHV1-2 HCs were comparable to what was observed in a Sanger sequencing-based study (67 and 33%, respectively)^36^. Many IGKV VRC01-class precursors had the VRC01-class bnAb signature LCDR3 sequence of QQYXX and E/N/Q at position 4 (Fig. 1j). The paired sequences of VRC01-class naive B cells isolated from donors 1–3 are provided in Supplementary Table 1. The precursor frequency of VRC01-class naive B cells, defined as the proportion of IGHV1-2 HC paired with a LC with a 5-AA LCDR3 among total naive B cells, ranged between 1 in 0.14 million to 0.28 million B cells (Table 2), similar to the previously reported frequency of 1 in 0.3 million B cells^36^.

**Table 2:** VRC01-class naive B cell precursor frequency calculated for cells sorted with eOD-GT8 tetramers.

	Donor 1	Donor 2	Donor 3
IGHV1-2 allele (presumed)^a^	*04/*04^b^	*02/*04	*02/*04
Total IgG^neg^ B cells screened	72.3 million	23.4 million	37.3 million
# eOD-GT8^++^eOD-GT8^KOneg^ cells identified	2129	708	1204
# Cells sorted	815	417	772
Sort recovery rate % (correction factor)	38.3% (2.6)	59.3 % (1.7)	64.1% (1.6)
# of obtained HC-LC sequence pairs	163	81	114
10× Genomics sequence recovery rate % (correction factor)	20% (5)	19.4% (5.1)	15.8% (6.8)
# of VRC01-class naive B cells	20	20	22
VRC01-class naive B cell frequency	1 in 3.6 million	1 in 1.2 million	1 in 1.7 million
Corrected frequency	1 in 0.28 million	1 in 0.14 million	1 in 0.16 million

^a^Presumed IGHV1-2 genotype based on BCR sequencing.

^b^This donor was directly sequenced to determine their IGHV1-2 alleles.

The LC variable gene usage among known VRC01-class antibodies allows room for diversity, but it is desirable for the germline encoded LCDR1 to be short in length or rich in flexible glycine or serine residues, as this region of the antibody is critical in accommodating the conserved N276 N-linked glycan near the CD4bs^39^. The majority of VRC01-class bnAbs utilize IGK, possibly because the average length of CDRL1 among IGK variable (IGKV) genes (6-AA) of circulating naive B cells in the human repertoire are shorter than IGL variable (IGLV) genes (9-AA)^36^. In sequences from 3 donors, 70–95% of VRC01-class BCRs possessed IGKV genes expressed by known VRC01-class bnAb subtypes (VRC01-subclass: IGKV3-20^39^; PCIN64-subclass: IGKV1-5^6^; N6-subclass: IGKV1-33^46^; VRC23-subclass: IGKV3-15^47^). BCRs expressing IGKV1-12 and IGKV1-39, not present in our previous studies^23,36^, were observed. Almost all of the IGKV sequences not observed among known VRC01-class bnAbs had glycine and serine containing 6 or 7-AA LCDR1s (Fig. 1k).

Of the few IGLV VRC01-class naive B cell sequences, the average LCDR1 length was 9-AA, in accordance with the average LCDR1 length of human IGLV genes (Fig. 1k). Notably, we identified two IGLV2-14 clones from two independent donors, representing VRC01-class precursor naive B cells belonging to the VRC-PG19 bnAb subclass^9,36^.

Previously it was shown that IOMA-class B cells could be isolated using eOD-GT8 tetramer probes^36^. IOMA is a CD4bs bnAb that has a IGHV1-2 HC but utilizes a IGLV2-23 LC with an 8-AA LCDR3 and a slightly different mode of binding compared to classic VRC01-class bnAbs^48^. In our current study, 5 IOMA-class B cells were obtained from two donors using tetramer probes (Fig. 1l). Overall, these combined results demonstrate that droplet scRNA-seq can be a productive approach to identify BCRs of vaccine-specific naive B cells.

### High-avidity antigen probes increase capture of off-target B cells

**Fig. 2:**
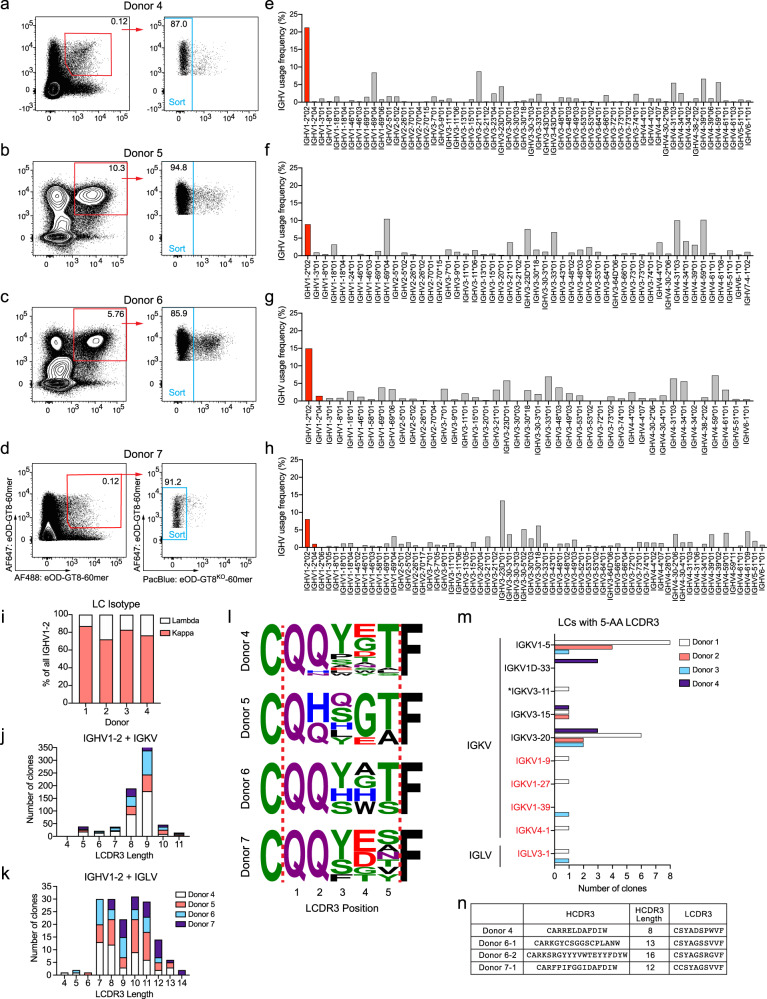
eOD-GT8-60mer-sorted VRC01-class naive B cells. B cells from healthy donor PBMCs were stained with eOD-GT8-60mer probes directly conjugated with fluorophores and sorted for IgG^neg^/eOD-GT8-60mer^++^ eOD-GT8^KO^-60mer^neg^ B cells, followed by BCR sequencing. **a**–**d** Flow cytometry of sorted cells in donor 4 (**a**), donor 5 (**b**), donor 6 (**c**), and donor 7 (**d**). **e**–**h** IGHV gene usage distribution of paired IgG^neg^/eOD-GT8-60mer^++^ eOD-GT8^KO^-60mer^neg^ B cells from donor 4 (**e**), donor 5 (**f**), donor 6 (**g**), and donor 7 (**h**). Bars corresponding to IGHV1-2 genes are colored in red. **i** Isotype distribution of LCs coexpressed with IGHV1-2. **j** LCDR3 lengths of IGKV LCs coexpressed with IGHV1-2. **k** As in **h**, but for IGLV LCs. **l** LCDR3 sequences of naive 5-AA IGKV LCs. **m** IGKV and IGLV usage distribution among eOD-GT8-60mer^++^ eOD-GT8^KO^-60mer^neg^, and 5-AA LCDR3 VRC01-class naive B cells. Text color coding is as in Fig. 1k. IGKV3-11 indicated by an asterisk has not been directly observed in VRC01-class bnAbs, although the LC of VRC01 was originally annotated as IGKV3-11^37^ due to its high similarity to IGKV3-20. **n** IOMA-class B cells sorted with eOD-GT8-60mer probes.

**Fig. 3:**
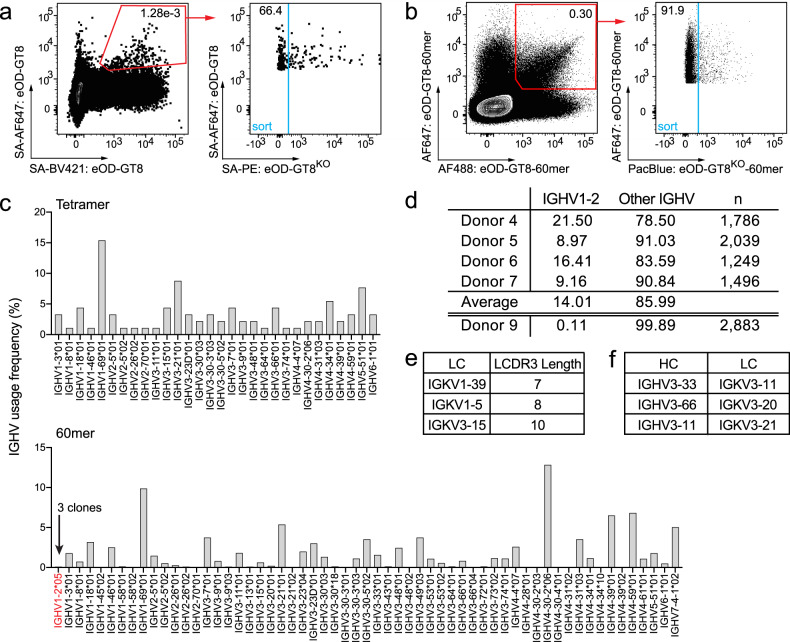
Some individuals do not have VRC01-class naive B cells due to incompatible IGHV1-2 alleles. B cells were enriched from donor 9 PBMCs, stained with eOD-GT8 tetramer or eOD-GT8 60mer probe set, and sorted for IgG^neg^/eOD-GT8^++^eOD-GT8^KOneg^ B cells. **a**-**b** Flow cytometry of IgG^neg^/eOD-GT8^++^eOD-GT8^KOneg^ cells using tetramer probes (**a**) or with (**b)** 60mer probes. Cells in the blue gate were sorted. **c** IGHV gene usage among paired BCR sequences derived from tetramer (upper, *n* = 91) and 60mer (lower, *n* = 2856) sorted cells. **d** Frequency of IGHV1-2 BCRs observed in eOD-GT8-60mer-sorted datasets in this study. **e** Characteristics of LCs paired to the three IGHV1-2*05 clones detected from 60mer-sorted cells. **f** Three 5-AA LCDR3 harboring sequences were found among 60mer-sorted cells from donor 9. None of the paired HCs were IGHV1-2.

Binding of low-affinity B cells to antigens can be dramatically augmented by using multimeric proteins to improve avidity^16,29,49–51^. However, the efficiency in isolating antigen-specific naive B cells by high-avidity nanoparticle probes is unknown. To probe the eOD-GT8-60mer^++^ eOD-GT8^KO^-60mer^neg^ naive B cell repertoire, we performed BCR sequencing of cells from four additional healthy donors sorted using eOD-GT8-60mer and eOD-GT8^KO^-60mer probes (Fig. 2). Cells were stained in two different ways. PBMCs from donors 4 and 7 were first enriched for B cells, then stained with fluorescent probes and antibodies as was done for all previous tetramer probe experiments (Fig. 2a, d). For donors 5 and 6, total PBMCs were instead first incubated with AlexaFluor647-conjugated eOD-GT8-60mer, then enriched for AlexaFluor647^+^ cells followed by staining with AlexaFluor488: eOD-GT8-60mer, Pacific-Blue: eOD-GT8^KO^-60mer, and antibodies (Fig. 2b, c). By doing so, percentage of eOD-GT8-60mer^++^ of IgG^neg^ B cells were enriched ~45-fold (Fig. 2a–d, Fig. 3b, and Supplementary Fig. 2a). Regardless of the sample preparation method used, a substantially larger fraction of IgG^neg^ B cells stained eOD-GT8-60mer^++^ eOD-GT8^KO^-60mer^neg^ than when tetramers were used. As a result, a much higher total number of naive B cells were sorted per donor (Table 3). More than 1000 paired BCR sequences were obtained from each donor.

**Table 3:** VRC01-class naive B cell precursor frequency calculated for cells sorted with eOD-GT8-60mers.

	Donor 4	Donor 5	Donor 6	Donor 7^c^
IGHV1-2 allele (presumed)^a^	*02/*04	*02/*02	*02/*04	*02/*04
Total IgG^neg^ B cells screened	25.6 million	246,890	419,167	5.32 million
Total B cells corrected for Ax647 enrichment^b^	N/A	11.0 million	18.7 million	N/A
# eOD-GT8^++^eOD-GT8^KOneg^ cells identified	27,902	24,054	20,737	3710
# Cells sorted	16,076	11,132	8727	5526
Sort recovery rate % (correction factor)	57.6% (1.7)	46.3 % (2.2)	42.1% (2.4)	N/A
# of obtained HC-LC sequence pairs	1786	2039	1249	1496
10× Genomics sequence recovery rate % (correction factor)	11.1% (9)	18.3% (5.5)	14.3% (7.0)	27.1% (3.7)
# of VRC01-class naive B cells	20	7	5	7
VRC01-class naive B cell frequency	1 in 1.28 million	1 in 1.57 million	1 in 3.74 million	1 in 0.75–1.32 million
Corrected frequency	1 in 0.08 million	1 in 0.13 million	1 in 0.22 million	1 in 0.21–0.38 million

^a^Presumed IGHV1-2 genotype based on BCR sequencing. Donor 5 may have a second allele other than *02 not identified by the sorted B cells.

^b^For donors 5 and 6, B cells were not stained prior to AlexaFluor647 enrichment. Therefore, we cannot back-calculate the true total number of IgG^neg^ B cells for these samples. Since the majority of cells bound to eOD-GT8-60mer probes were not VRC01-class antibodies, we estimated the normal proportion of B cells that are eOD-GT8-60mer^++^ by averaging frequencies from 60mer-sorted samples: donors 4 and 7–9 (Table 1). Frequency of B cells that are eOD-GT8-60mer^++^ eOD-GT8-60mer^+^ post-AlexaFluor647 enrichment was estimated by averaging values from 60mer donors. Average post-enrichment frequency was divided by pre-enrichment frequency to yield 44.61-fold enrichment of B cells.

^c^In donor 7, not all sorted events were recorded during acquisition. Sort recovery rate correction factor was not applied in calculating the corrected precursor frequencies. An estimation of the precursor frequency range is provided. See “Methods” section for detail.

Relative IGHV1-2 gene usage among eOD-GT8-60mer^++^ eOD-GT8^KO^-60mer^neg^ B cells in each donor ranged from 9 to 22%, compared to 32–60% when using tetramer probes (Figs. 1d–f and 2e–h). The sequences of paired VRC01-class naive B cells isolated from donors 4 to 7 are provided in Supplementary Table 2. Of the IGHV1-2^+^ B cells, the ratio of IGKV to IGLV were similar to tetramer-sorted BCRs (Fig. 2i), but only 2.4–8.6% of IGKV and 0–2.9% of IGLV BCRs possessed 5-AA LCDR3s regardless of the LC isotype (Fig. 2j, k). Single-cell Sanger sequencing of eOD-GT8-60mer^++^ eOD-GT8^KO^-60mer^neg^ B cells in another donor (donor 8) also found low frequencies of IGHV1-2 HCs, of which only one was paired with a 5-AA LCDR3 LC (Supplementary Fig. 2a–c). Thus, the difference in VRC01-class BCR sequence recovery rates between cells sorted using tetramer and 60mer probes in this study was not an artifact associated with the sequencing method. All observed VRC01-class naive B cell sequences among eOD-GT8-60mer-bound B cells had features reminiscent of VRC01-class bnAbs (Fig. 2l, m). Interestingly, histidine residues were frequently observed within the LCDR3 of the few sequences isolated from donors 5 and 6. In previous studies, monomeric binding of eOD-GT8 to naive VRC01-class antibodies was determined for eOD-GT8 sorted human naive B cells^23,36^. Within this dataset, expressed antibodies with histidine containing LCDR3s had dissociation constants (*K*_D_) within the range of antibodies without LCDR3 histidine residues (Supplementary Fig. 2d). Three IOMA-class B cells were also found among B cells sorted from donors 4, 6, and 7 (Fig. 2n). These data suggest that the eOD-GT8-60mer binds rare VRC01-class naive B cells as designed, but the high avidity of the antigen captures a more diverse population of B cells much more so than tetramers. Concordant with this observation, the final calculated VRC01-class B cell precursor frequencies identified by eOD-GT8-60mer probes ranged between 1 in 0.08 and 0.38 million naive B cells, a range similar to the precursor frequency determined using eOD-GT8 tetramer probes (Table 3)^23,36^.

### IGHV1-2*05 is unable to bind the HIV CD4bs in a VRC01-like manner

During our study, we identified one donor (donor 9) who had approximately tenfold lower eOD-GT8^++^eOD-GT8^KOneg^ naive B cells identified using tetramers, compared to previous donors (Figs. 1a–c and 3a). We sequenced tetramer-sorted eOD-GT8^++^eOD-GT8^KOneg^ B cells by droplet scRNA-seq and found that, surprisingly, none of BCRs expressed the IGHV1-2 gene (Fig. 3c). When eOD-GT8-60mer nanoparticles were used as probes to stain cells from donor 9, the frequency of eOD-GT8-60mer^++^ eOD-GT8-60mer^KOneg^ naive B cells were similar to what was observed in other donors from whom we were able to isolate VRC01-class naive B cells (Fig. 2a–d, Fig. 3b, and Supplementary Fig. 2a, ref. ^36^). Nearly 3000 paired BCR sequences were obtained from the 60mer-sorted B cells from donor 9, but only three cells expressed an IGHV1-2 gene (Fig. 3c, d) and none of the three IGHV1-2^+^ B cells coexpressed a LC with a 5-AA LCDR3 (Fig. 3e). Likewise, HCs paired with the few 5-AA LCDR3 LCs observed in the dataset did not express IGHV1-2 (Fig. 3f). The three IGHV1-2 sequences were annotated as the *05 allele, which is predicted to be unsuitable as a VRC01-class precursor due to a missing germline encoded W50 residue that forms a conserved interaction with N280 of gp120 in all VRC01-class bnAbs^16,38,40^. We therefore hypothesized that this donor had two IGHV1-2 alleles with reduced potential to develop VRC01-class bnAbs. The IGHV1-2 genotype of donor 9 was next determined to be IGHV1-2*05/*05 by targeted PCR, cloning, and Sanger sequencing (Supplementary Fig. 3a). Thus, both single-cell sorting and bulk sequencing as well as genotyping identify this subject as an individual with a B cell repertoire incompatible with eOD-GT8 binding due to a missing IGHV1-2 W50^16,38,40^.

### Precursor frequencies of eOD-GT8-binding IGHV1-2 naive B cells are affected by allelic variation

**Fig. 4:**
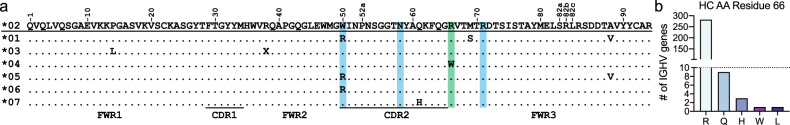
Allelic variations in IGHV1-2 genes impact CD4bs recognition and possibly BCR expression. **a** Amino acid sequence alignment of the seven annotated IGHV1-2 alleles. The IGHV1-2*02 allele is shown as the reference. The Kabat numbering scheme of the residues is shown. Residues highlighted in blue indicate the position of key residues in IGHV1-2 required for binding to the CD4bs. Residue position 66 is shown in green. **b** Of all the IMGT curated functional IGHV genes (*n* = 296), the majority of the genes utilize an arginine residue at position 66.

In addition to the effects of *05 allele, we also observed differences in IGHV1-2^+^ B cell frequencies associated with other IGHV1-2 allelic variants. Notably, among our tetramer donors, donor 1 with the lowest frequency of IGHV1-2^+^ B cells (32%) was homozygous for the IGHV1-2*04 allele (Fig. 1d and Supplementary Fig. 3b). The VRC01-class naive B cell precursor frequency in this donor was lower by ~2-fold, 1 in 0.25 million compared to an average of 1 in 0.15 million naive B cells in donors 2 and 3 (Table 2), who were both *02/*04 heterozygotes. Interestingly, within donors 2 and 3, we also observed that most of the identified IGHV1-2 BCRs utilized the *02 allele (Fig. 1e, f). Similar allele usage patterns were observed among 60mer-sorted B cells from donors 4 to 7 (Fig. 2e–h). Of the 7 curated IGHV1-2 alleles in IMGT, all currently known VRC01-class bnAbs are thought to derive from the IGHV1-2*02 allele^6,39,40^. The *02, *03, *04, and *07 alleles have germline encoded W50, N58, and R71 residues (Kabat numbering) required for VRC01-like CD4bs recognition^16,40^ (Fig. 4a). It is likely that *01 and *03 alleles are old sequencing artifacts^52^, explaining why the *03 allele is yet to be observed among VRC01-class B cells. The *07 allele has not been observed in donors so far likely due to rarity, as the *07 allele was only recently annotated (GenBank: MN337615)^53^. The *04 allele is distinct in that it encodes a W66 in framework region (FWR) 3, in place of an arginine found in other IGHV1-2 alleles. Arginine is the preferred residue at position 66 among all annotated functional human IGHV genes (Fig. 4b). The next most common variants in this position are Q66 and H66, which both retain polar side chains. We speculate that the hydrophobic tryptophan residue exposed on the surface of an IGHV1-2*04-encoded antibody may impact the solubility of the BCR, thereby affecting development of IGHV1-2*04 B cells. The *06 allele is represented by an arginine at AA position 50, analogous to the *05 allele, which may hamper potential of IGHV1-2*06-encoded BCRs to become VRC01-class bnAbs. In light of the findings above with respect to IGHV1-2*04, *05, and *06, we sought to explore IGHV1-2 allele signatures at the population level.

### IGHV1-2 allele frequency variation among human ethnic groups

**Fig. 5:**
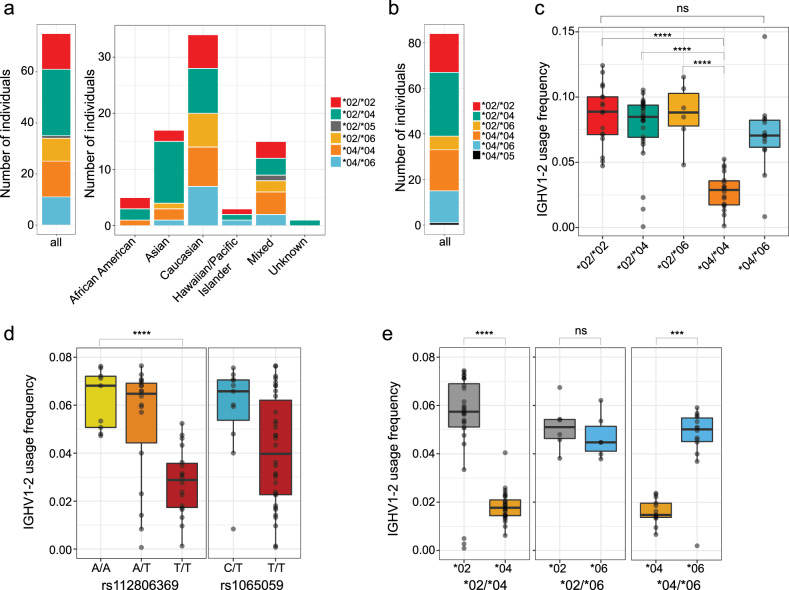
Population-level IGHV1-2 genotype frequencies and allele-specific effects on naive antibody repertoire usage. **a** Number of individuals of each inferred IGHV1-2 genotype in the collective cohort (left) and within each population subgroup (right), based on allele inferences made using RNA-seq data (*n* = 75 donors). **b** Number of individuals of each IGHV1-2 genotype based on inferences made from IgM/IgD expressed antibody RepSeq data (*n* = 84 donors). **c** IGHV1-2 gene usage within the IgM/IgD repertoire partitioned by IGHV1-2 genotype (*n* = 83 donors). **d** IGHV1-2 gene usage in the IgM/IgD repertoire partitioned by genotypes at SNPs rs112806369 and rs1065059 (*n* = 83 donors). **e** IGHV1-2 allele-specific usage within the IgM/IgD repertoire in IGHV1-2 heterozygotes (*02/*04, *n* = 28 donors; *02/*06, *n* = 6; *04/*06, *n* = 14). All boxplots are plotted as follows: center line, median; box limits, upper and lower quartiles; whiskers, 1.5× interquartile range; points outside the whiskers, outliers. One-way ANOVA: ***P < 0.0001; ****P < 0.00001; ns not significant.

To survey inferred allele and genotype frequencies at single-nucleotide polymorphisms (SNPs) within IGHV1-2 among different population subgroups, we first leveraged naive B cell-derived transcriptomic data from the DICE cohort (database of immune cell expression, expression quantitative trait loci, and epigenomics)^54^, representing an ethnically diverse collection of donors (*n* = 75; African American, *n* = 5; Asian, *n* = 17; Caucasian, *n* = 34; Hawaiian/Pacific Islander, *n* = 3; Mixed ethnicity, *n* = 15; Unknown, *n* = 1). We focused our analysis on three primary SNPs (rs1065059, rs112806369, and rs12588974) within this dataset, which differentiate IGHV1-2 alleles *02, *04, *05, and *06 (Fig. 5a; Supplementary Fig. 4). Using all individuals within this cohort with sufficient RNA-seq reads mapping to these positions, we inferred SNP genotypes (Supplementary Fig. 4) and IGHV1-2 allele-based genotypes (Fig. 5a and Supplementary Table 3). Based on allele inferences, *02, *04, and *06 alleles were observed at frequencies of 42, 43, and 13% (Supplementary Table 3). The *02/*02 (18.6%), *02/*04 (34.6%), and *04/*04 (18.6%) genotypes were most common, followed by *02/*06 (12%) and *04/*06 (14.6%) heterozygotes (Fig. 5a). Evidence for the *05 allele was limited (1/75 individuals), and no *05/*05 homozygotes were observed in this cohort (Fig. 5a). Similarly, we did not observe SNP alleles representing *01, *03, or *07 (Supplementary Fig. 4). It was notable that, in contrast to *05, both the *06 and *04 alleles were relatively prevalent in the population (Fig. 5a). Specifically, individuals lacking the *02 allele, represented by *04/*04 and *04/*06 genotypes, were observed at a collective frequency of ~33% in the overall cohort. Frequencies of these genotypes were moderately higher in both Caucasian (41%) and Mixed (40%) subgroups (Fig. 5a). In the eOD-GT8^++^eOD-GT8^KOneg^ BCR sequencing data, we observed that the *04 allele was associated with reduced VRC01-class B cell precursor frequencies relative to *02 among presumed *02/*04 heterozygotes (Figs. 1 and 2 and Tables 2 and 3). Therefore, the VRC01-class B cell priming efficacy in *04 individuals may be reduced relative to *02 allele harboring individuals.

### Allele frequencies at SNPs within IGHV1-2 vary between human population groups

Allele frequencies were next examined at each of the three key IGHV1-2 SNPs (rs1065059, rs112806369, and rs12588974) in data from the 1000 Genomes Project (1KGP^55^), which had been done previously when less data were available^16^. IGHV1-2 SNP allele frequency biases were observable among human subpopulations (Supplementary Fig. 5). While technical confounding factors related to the use of short-read mapping and cell-line artifacts are known to influence the accuracy of genotype frequencies in the 1KGP dataset^28,56^ requiring that these data be interpreted with caution, the data are consistent with other sources we considered. For example, consistent with the DICE cohort studied above, the 1KGP dataset also provided evidence that minor alleles at two SNPs associated with non-*02 alleles (rs112806369, *04; rs1065059, *05/*06) are relatively common across populations (14.9–46.4%). In comparison, while the SNP allele representing valine at position 86, observed in alleles *01 and *05 (rs12588974), occurs at lower frequencies in most populations (2.6–11%), it appears to be more common in the East (38.6%) and South Asian (22.6%) subpopulations (Supplementary Fig. 5). The fact that this contrasts with the limited support for *05 in the RNA-seq dataset analyzed here could be explained by the smaller population subgroup sizes, as well as known expression biases in *05^57^ that may make it more difficult to detect from RNA-seq data. These observations warrant more comprehensive sequencing of IGHV1-2 as a means to fully clarify the extent of population-level germline variation at this locus.

### Variable IGHV1-2 usage in the naive IgM/IgD repertoire is associated with distinct alleles

Previous reports have identified allele-associated IGHV gene usage differences within naive and antigen-stimulated B cell repertoires^57–61^. Given this, and the apparent preferential selection of the IGHV1-2*02 allele observed in our V(D)J sequencing data, we next investigated whether skewed IGHV1-2 allele usage patterns were observable within the naive repertoire at the population level. To do this, we utilized a separate publicly available IgM/IgD expressed repertoire sequencing (RepSeq) dataset from a cohort of healthy donors (*n* = 84)^62,63^. Consistent with genotype data reported above, among the individuals included in the RepSeq dataset, we observed the presence of IGHV1-2*02, *04, and *06 alleles at the highest frequencies (Supplementary Table 3), represented by *02/*02 (20.2%), *04/*04 (21.4%), *02/*04 (33.3%), *02/*06 (7.1%), and *04/*06 (16.6%) genotypes (Fig. 5b). Only one heterozygous subject carried the *05 allele (*04/*05). Again, the apparent low prevalence of *05 may be due to low usage of this allele^57^; the usage frequency observed in the *04/*05 individual was 1.3%. We observed a range in IGHV1-2 usage frequencies across the remaining subjects (0.06–14%), which we found to associate with allelic variation (one-way analysis of variance (ANOVA), effect of genotype, *P* = 3.76e−10; Fig. 5c). Consistent with observations discussed in above sections, *04/*04 homozygotes had the lowest IGHV1-2 usage relative to all other genotypes (one-way ANOVA, *P* = 2.14e−10, vs. *02/*02; *P* = 2.82e−09, vs. *02/*04; *P* = 1.86e−07, vs. *02/*06; Fig. 5c). Usage in the *04/*06 heterozygotes, however, was not significantly different from *02/*02 (one-way ANOVA, *P* = 0.12). This effect of *04 on IGHV1-2 usage was clearly observable when we grouped subjects by genotypes at SNP rs112806369 (Fig. 5d), which differentiates *04 (T) from *06 and *02 (A) alleles. Individuals of the A/A and A/T genotypes had higher IGHV1-2 usage than those of the T/T genotype (one-way ANOVA, *P* = 1.46e−11; Fig. 5d). In contrast, partitioning subjects by genotypes at SNP rs1065059, which differentiates *06 (C) from *04 and *02 (T), did not reveal significant differences (one-way ANOVA, *P* = 0.21; Fig. 5d). Consistent with earlier observations^60,61^, we also observed *04 allele-specific usage biases within IGHV1-2 heterozygotes (Fig. 5e). In individuals of *02/*04 and *04/*06 genotypes, *04 usage was significantly lower than that of the *02 (one-way ANOVA, *P* = 6.65e−12) and *06 alleles (one-way ANOVA, *P* = 0.0003). This contrasted with allele-specific patterns in *02/*06 heterozygotes, in which both alleles were used at comparable frequencies (one-way ANOVA; *P* = 0.47). These results implied that the *02 usage bias relative to other IGHV1-2 alleles among VRC01-class bnAbs and naive B cells likely occurs due to genetic impacts on V(D)J recombination frequencies and/or BCR expression.

## Discussion

Naive precursor B cells to different antigens can be identified by using fluorescent GT probes such as eOD-GT8, coupled with single-cell Sanger sequencing^23,36^. Using a bulk-sort based, high-throughput single-cell sequencing technology, we have confirmed that the human B cell repertoire can also be screened for rare antigen-specific naive B cells with droplet-based sequencing. The VRC01-class naive B cell frequencies calculated based on sequences derived from the 10× Genomics Chromium platform were comparable to previous numbers determined by Sanger sequencing. In this study, we used two different probes: SA tetramers and 60mer nanoparticles. Regardless of the probe used, the final calculated precursor frequencies of naive VRC01-class B cells identifiable by eOD-GT8 were similar.

The eOD-GT8-60mer probes were found to be less efficient at enriching for VRC01-class naive B cells than tetramer probes. The majority of 60mer-sorted IGHV1-2^+^ B cells were not paired with LCs with 5-AA LCDR3s. Compared to the tetramer, the 60mer probe was also less selective for IGHV1-2 overall. Some of these BCRs identified by the 60mer probe, particularly those that are IGHV1-2^+^, may have low monovalent affinity to the CD4bs that was enhanced by avidity. We previously found that non-VRC01 class IGHV1-2 BCRs and non-IGHV1-2 BCRs expressed from naive B cells isolated by tetramer probes had relatively weak average affinities^36^. In mice, high-valency eOD-GT-60mer immunogens recruited a large number of low-affinity non-VRC01-class B cells into germinal centers^64^. This was in contrast to eOD-GT tetramer immunogens, for which high-affinity B cells were preferentially recruited. That in vivo finding is consistent with the screening results here, wherein the proportion of VRC01-class precursor B cells among eOD-GT8-60mer^++^ eOD-GT8KO-60mer^neg^ B cells was reduced compared to tetramer-sorted cells, but the absolute precursor frequency of VRC01-class naive B cells calculated using either method was comparable. Overall, our results here imply that using tetramer probes coupled with 10× Genomics Chromium technology would be the most effective way to examine the B cell repertoire, although high-avidity nanoparticle probes hold the potential for detecting low-affinity B cells if future germline-targeting programs require isolation of particularly low-affinity naive B cells.

We observed that not all IGHV1-2 germline alleles appear to make equal contributions to the circulating VRC01-class B cell precursor pool, consistent with previously published work^40^. Specifically, we showed that the presence of the IGHV1-2*02 allele within an individual’s genotype was associated with higher numbers of VRC01-class B cells. This was particularly true when comparing individuals harboring an *02 allele, compared to those with *04/*04 and *05/*05 genotypes. A twofold reduction in VRC01-class precursors was observed in the *04/*04 donor relative to those with an *02 allele. A complete absence of eOD-GT8-binding IGHV1-2 BCRs was observed in an *05/*05 donor. Further, in four heterozygous *02/*04 individuals, we observed that eOD-GT8-binding B cells were overwhelmingly associated with the use of IGHV1-2*02-derived BCRs. We noted that, among all curated IGH alleles in IMGT, IGHV1-2*04 is one of the few IGHV alleles not encoding an arginine at AA position 66 and the only allele encoding a tryptophan at this position. It is plausible that W66 has potential functional consequences for BCR expression or solubility. It remains unclear whether the reduced precursor frequencies resulting from the *04 allele will impact the outcome of VRC01-class B cell priming immunizations.

Interestingly, mirroring observations from our single-cell BCR analyses, we also noted strong genetically driven usage biases of IGHV1-2 alleles in the naive repertoire of an expanded cohort of healthy donors. These analyses showed that, while *02 usage was relatively high in the overall naive repertoire, the *04 allele was utilized at very low frequencies in both homozygous and heterozygous individuals. In addition, we found by BCR RepSeq that the *06 allele was utilized at relatively high frequency within the naive repertoire, at levels comparable to *02. Because the *06 allele lacks the critical W50 residue present in *02, it is predicted to not contribute to VRC01-class antibodies. Whether *02/*06 or *04/*06 heterozygosity impacts the frequency of VRC01-class B cell precursors should be directly tested in future studies.

Together, these data indicated that inter-individual variation in GT vaccine responses, driven by differences in IGHV1-2 genotype, could be expected. With this in mind, we investigated the frequencies of IGHV1-2 alleles and genotypes at the population level. Principally, this analysis revealed that both *04 and *06 alleles are frequent, and individuals lacking IGHV1-2*02 in their genomes make up a significant fraction of the population. In particular, the distribution of IGHV1-2 alleles stratified by ethnic groups revealed differences that should likely be considered when developing vaccines. However, we note that the majority of the primary cohort studied here was of Caucasian and Asian/Pacific Islander descent, and the true allelic distribution may be different within a larger cohort comprised of relatively equivalent numbers of individuals of all ethnicities; analysis of data from the 1KGP provided some evidence for this.

In summary, we emphasize that a primary consideration in developing germline-targeting vaccines should be whether B cells that are to be targeted by immunogens exist within the naive B cell repertoire and whether those B cells occur at a high enough precursor frequency. Better understanding of the factors that contribute to variation in naive B cell precursor frequencies and repertoires will be critical moving forward. As illustrated in this study, the antigen-specific naive B cell repertoire can be examined relatively quickly with state-of-the-art sequencing technologies.

## Methods

### Probe preparation

Avi-tagged eOD-GT8 and eOD-GT8^KO^ monomers, and eOD-GT8-60mer and eOD-GT8^KO^-60mer nanoparticles were recombinantly expressed in HEK293F cells by transient transfection and purified as summarized elsewhere^16^. The eOD-GT8^KO^ probes are eOD-GT8^KOII^ probes described in our previous study^36^, renamed for simplicity. Avi-tagged monomer probes were biotinylated and purified as previously described^32^. To generate eOD-GT8 tetramer probes, biotinylated monomers were mixed with fluorescently labeled streptavidin (SA-Alexafluor647 or SA-Brilliant Violet 421) at a molar ratio of 4 monomers: 1 SA, in a stepwise manner. One-third of the total amount of SA was added to the biotinylated probes and incubated for 20 min in the dark at room temperature, and the process was repeated twice. The KO probe, eOD-GT8^KO^:SA-phycoerythrin (PE) was prepared in the same manner. eOD-GT8-60mer nanoparticles were directly labeled with fluorophores using AlexaFluor488 or AlexaFluor647 protein labeling kits (Life Technologies) according to instructions supplied by the manufacturer. eOD-GT8^KO^-60mer nanoparticles were labeled with the Pacific Blue Protein Labeling Kit (Life Technologies).

### Sorting and 10× Genomics V(D)J sequencing

Buffy coats were obtained from healthy donor blood samples from the San Diego Blood Bank from consenting participants, in accordance with protocols approved by the La Jolla Institute for Immunology (LJI) Institutional Review Board. PBMCs were isolated from blood by the LJI Blood Processing Core. Frozen PBMCs isolated from blood were thawed and recovered in R10 (RPMI, 5% fetal bovine serum, 1× PenStrep, 1× Glutamax) and stained for sorting as previously described^36^. In brief, total PMBCs were enriched for B cells using CD19 MicroBeads (Miltenyi Biotec). Purified B cells were enumerated and stained for 20 min at 4 °C with a mix of tetramer or 60mer probes (two eOD-GT8 probes and one eOD-GT8^KO^ probe) in R10. For donor 7, purified B cells were first stained with PacificBlue: eOD-GT8^KO^-60mer for 20 min at 4 °C, followed by the addition of AlexaFluor647: eOD-GT8-60mer and AlexaFluor488: eOD-GT8-60mer for 20 min at 4 °C. Without washing, antibody master mix was added to the cells for an additional 20 min at 4 °C. Cells were washed twice and passed through a 70 μm mesh filter prior to sorting.

Where indicated, total PMBCs were stained with Alexafluor647: eOD-GT8-60mer for 20 min at 4 °C. Cells were washed twice, then positively selected for AlexaFluor647^+^ cells using Anti-Cy5/Anti-AlexaFluor647 MicroBeads (Miltenyi Biotec). The AlexaFluor647-enriched cells were stained for 20 min at 4 °C with AlexaFluor488: eOD GT8-60mer and PacificBlue: eOD-GT8^KO^-60mer. Without washing, antibody master mix was added for 20 min at 4 °C. Cells were washed twice and passed through a 70 μm mesh filter prior to sorting.

All samples were sorted on a BD FACSAria II sorter using an 85 μm nozzle. eOD-GT8^++^eOD-GT8^KOneg^ cells were gated as lymphocytes/singlets/dump^neg^ (anti-CD14, CD16, CD3, IgG) Live/Dead^neg^/CD19^+^ or CD20^+^/eOD-GT8^+^eOD-GT8^+^/eOD-GT8^+^eOD-GT8^KOneg^ and bulk sorted into a 1.6 mL Eppendorf tube containing 50 μL of R10 catch buffer. For donor 7, IgG was stained with a separate color and dump^neg^ Live/Dead^neg^ cells were followed by an IgG^neg^ CD20^+^ gate. In some experiments, the same fluorochrome channel was used for dump and Live/Dead. Antibodies and dilutions used are as follows: CD3 (UCHT1, eBiosciences cat# 47-0038-42, APC-e780, 1:100), CD14 (M5E2, Biolegend cat# 301820, APC-Cy7, 1:100), CD16 (CB16, eBiosciences cat# 47-0168-42, APC-e780, 1:100), CD19 (HIB19, eBiosciences cat# 25-0199-42, PE-Cy7 1:100), CD20 (2H7, eBiosciences, PE-Cy7, 1:100), IgG (HP6019, Biolegend cat# 490314, APC-Cy7, 1:100), and IgG (G18-145, BD Biosciences cat# 563247, BV510, 1:100). In some cases, TotalSeq-C anti-human Hashtag antibodies (LNH-94 and 2M2, Biolegend) were used to multiplex samples from different donors and individually added along with the master mix at a concentration of 0.1 μg per million B cells.

Sorted cells were spun down for 5 min at 500 × *g*, and extra buffer was removed until only approximately 30 μL (or 40 μL for v2 chemistry) was remaining in the tube. The pelleted cells were resuspended in 30 μL and prepared following instructions provided for Chromium Single Cell V(D)J Reagent Kits with Feature Barcoding Technology (10× Genomics). The legacy system was used for all but one experiment (donor 7) performed in this manuscript. V(D)J cDNA libraries were sequenced on an Illumina MiSeq or NovaSeq 6000 using a 150×150 bp configuration, aiming for ~5000 read pairs per cell. Where hashtag feature barcoding antibodies were used, hashtag cDNA libraries were sequenced on the NovaSeq 6000 using the same configuration as the V(D)J library. Target number of hashtag reads was ~1600 read pairs per cell, amounting to approximately 1:3 hashtag: V(D)J library pooling ratio. For donor 7, the Single Cell Immune Profiling v2 Chemistry Kit with dual indexing was used for library preparation and sequenced using a 26 × 8 × 0 × 91 configuration as suggested by the manufacturer.

### BCR sequence analysis

The sequenced V(D)J contigs were assembled and annotated using CellRanger V(D)J within the CellRanger software packages v3.1 (all donors except donor 7) and v4.0 (donor 7) (10× Genomics), using an IG reference library compiled from IMGT references. Each given cell barcode was associated with its productive HC and LC information. First, cells associated with unpaired HC or LC contigs were removed from the dataset. Next, cell barcodes associated with multiple HC contigs were eliminated as this indicated that more than one cell was captured within a droplet. Barcodes with more than one LC contig of the same isotype were removed for the same reason. For cell barcodes that expressed one HC contig with one IGK and one IGL contig, it was assumed that the HC would be paired with the IGL LC, because IGL rearranges when IGK cannot be co-expressed with the HC. In all the samples, some proportion of paired BCRs were annotated as expressing class-switched isotypes. All cells other than those annotated as expressing an IgM or IgD isotype HC were excluded from analysis.

Where relevant, hashtag reads were enumerated using CellRanger count. Hashtag counts were associated with productive assembled V(D)J sequences based on cell barcodes, and the information was compiled into a single file in a tabular format. Hashtagged samples were deconvoluted based on the following hashtag read count criteria: the cell must have ≥1000 read pairs from its expected hashtag, while having <100 read pairs from all other hashtags. For example, if a cell was associated not only with 5000 hashtag-1 reads but also with 110 hashtag-2 reads, the cell was considered to be contaminated and excluded from analysis. The Python script used to generate the tabulated data is available on GitHub (https://github.com/LJI-Bioinformatics/Filter-Cellranger-VDJ).

Because CellRanger V(D)J failed to assign D-genes to some of the sequences, HC sequences were reanalyzed through IMGT/V-QUEST^65,66^, and additional filters were applied to demarcate naive VRC01-class B cells. Of all the identified sequences that had an IGHV1-2 HC paired with a LC with 5-AA LCDR3, HC nucleotide mutations were observed in three of the clones. These B cells were not considered to be naive. IGL contigs were also screened to identify mutations. One clone had a large mismatching chunk in its IGKV FWR1, possibly due to an assembly error. This clone was excluded from the final VRC01-class naive B cell count. Two clones had a single-nucleotide mutation in the IGKV gene but were not excluded because no mutations were detected in the junctional annotation or in the HC IGHV1-2 gene. Interestingly, among IGKV1-5*03 contigs, a G>A variation was observed at IGKV1-5 V-region residue position 153. The same variant was frequently observed among IGKV1-5 LCs in our previous naive VRC01-class datasets^36^. Thus, these IGKV1-5 paired BCRs were considered to be naive under the assumption that we may be observing evidence of an undocumented IGKV105 allele. In summary, of all the identified IGHV1-2^+^ 5AA-LCDR3 BCRs, 4 clones were excluded from our final list of naive VRC01-class B cells compiled in Supplementary Tables 1 and 2.

For donor 7, not all sorted events were recorded, resulting in a higher number of cells sorted than cells identified. Therefore, the total number of IgG^neg^ B cells screened in Table 3 refers to cells identified from acquired events. Since the number of eOD-GT8^++^eOD-GT8^KOneg^ cells identified was ~67% of total B cells that were sorted, we estimate that only ~4 VRC01-class naive B cells would have been recovered from donor 7 if 3710 cells were processed for sequencing. Using this assumption, the precursor frequency range prior to correction for loss in sequence recovery would be 4–7 VRC01-class naive B cells in 5.32 million cells.

### Sanger sequencing

The IGHV1-2 locus was PCR amplified from genomic DNA (25 ng) of each donor examined using the Qiagen HotStar HiFidelity Polymerase Kit (Catalog No. 202602), with previously published oligos (5’-GAGACTCTGTCACAAACAAACCA-3’; 5’-GTGTGTTCTCTTTCTCATCTTGGA-3’). Thermocycler conditions included an initial incubation at 95 °C for 5 min, followed by 30 cycles of: 94 °C for 15 s, 60 °C for 1 min, 72 °C for 1 min, and final extension at 72 °C for 10 min. The resulting PCR product was cloned using the TOPO™ TA Cloning™ Kit, with One Shot™ TOP10 Chemically Competent *E. coli* (Catalog Number K4575J10). Briefly, TOPO cloning reactions were prepared for each PCR product using the manufacturer’s protocol. Five colonies were selected for Mini-Prep (Catalog No. 27104), and extracted DNA was Sanger sequenced using T7 and SP6 oligos. Allele sequences were confirmed by visual inspection of sequence chromatograms (Supplementary Fig. 3).

### Population-level genotype analysis

Naive B cell RNA-seq were mapped to the hg19 reference genome using TopHap v1.4.1^67^ as part of a previously published study^54^. RNA-seq “.bam” files were obtained from this study, and the software package SAMtools^68^ was used to assess read depth and allele calls at SNPs representing each of the seven currently curated IGHV1-2 alleles (see Fig. 4). To infer alleles and genotypes at each position, we required a total read depth (>3) and allele-specific read depth >1; only base calls with quality scores >32 (Phred 66) were considered. Based on these filter criteria, only 75 individuals from this cohort had sufficient read data available. Only positions representing the *02, *04, *05, and *06 alleles exhibited variation between individuals (rs1065059, rs112806369, and rs12588974; Supplemental Fig. 3). IGHV1-2 allele-based genotypes were inferred based on combined genotype calls made at each of these three SNPs. Phase 3 variant call summary data from the 1KGP^55^ was obtained from the Ensembl genome browser (https://uswest.ensembl.org/).

### Population-level analysis of expressed IgM/IgG antibody repertoire sequencing data

We analyzed previously published naive antibody repertoire sequencing datasets from 84 healthy human donors, derived from PBMC RNA (SRA: SRP161839)^62,63^. For each sample in this dataset, we selected reads representing IgM/IgD isotypes, assigned these sequences to the closest germline IGHV and IGHJ genes, and computed CDR3s using the DiversityAnalyzer tool^69^. Germline genotypes for each sample, inferred by TIgGER^70^, were downloaded from VDJbase^63^. We excluded individuals in which >2 IGHV1-2 alleles were inferred. To minimize the impact of sequencing and amplification errors, we collapsed IgM/IgD sequences within an individual with identical CDR3s and computed usage frequency of IGHV1-2 as the number of collapsed sequences aligned to IGHV1-2 within each individual normalized by the total number of collapsed sequences; allele-specific usage frequencies in individuals heterozygous for IGHV1-2 alleles were computed in the same way. Because we used only IgM/IgD sequences, we considered that clonal expansion would have little to no effect on the data. Statistical associations between IGHV1-2 gene/allele usage and IGHV1-2/SNP genotypes were assessed using one-way ANOVA in R; the single *04/*05 individual was excluded from these analyses.

### Data analysis and visualization

Flow cytometric data were analyzed in FlowJo v10.5.3 (FlowJo). BCR sequencing data and allele analyses were performed as described in the above sections. Graphs were generated using Prism 8 (GraphPad) and R. Sequence Logos were generated by WebLogo 3^71^.

### Reporting summary

Further information on research design is available in the Nature Research Reporting Summary linked to this article.

## Supplementary information

Supplementary Information

Reporting Summary

## Data Availability

Heavy chain sequences shown in Supplementary Tables 1 and 2 have been deposited at GenBank with the accession codes MZ594686–MZ594887. Complete CellRanger V(D)J assembled FASTA files can be provided upon request.

## References

[1] Burton DR, Mascola JR (2015). Antibody responses to envelope glycoproteins in HIV-1 infection. Nat. Immunol..

[2] Burton DR, Hangartner L (2016). Broadly neutralizing antibodies to HIV and their role in vaccine design. Annu. Rev. Immunol..

[3] Landais E, Sok D (2021). Nature or nurture: factors that influence bnAb development. Cell Host Microbe.

[4] Doria-Rose NA (2014). Developmental pathway for potent V1V2-directed HIV-neutralizing antibodies. Nature.

[5] Landais E (2017). HIV envelope glycoform heterogeneity and localized diversity govern the initiation and maturation of a V2 apex broadly neutralizing antibody lineage. Immunity.

[6] Umotoy J (2019). Rapid and focused maturation of a VRC01-class HIV broadly neutralizing antibody lineage involves both binding and accommodation of the N276-glycan. Immunity.

[7] MacLeod DT (2016). Early antibody lineage diversification and independent limb maturation lead to broad HIV-1 neutralization targeting the Env high-mannose patch. Immunity.

[8] Soto C (2016). Developmental pathway of the MPER-directed HIV-1-neutralizing antibody 10E8. PLoS ONE.

[9] Zhou T (2013). Multidonor analysis reveals structural elements, genetic determinants, and maturation pathway for HIV-1 neutralization by VRC01-class antibodies. Immunity.

[10] Burton, D. R. What are the most powerful immunogen design vaccine strategies?: reverse vaccinology 2.0 shows great promise. *Cold Spring Harb. Perspect. Biol*. 10.1101/cshperspect.a030262 (2017). 10.1101/cshperspect.a030262 PMC5540812 28159875

[11] Xiao X (2009). Germline-like predecessors of broadly neutralizing antibodies lack measurable binding to HIV-1 envelope glycoproteins: implications for evasion of immune responses and design of vaccine immunogens. Biochem. Biophys. Res. Commun..

[12] Zhou T (2010). Structural basis for broad and potent neutralization of HIV-1 by antibody VRC01. Science.

[13] Scheid JF (2011). Sequence and structural convergence of broad and potent HIV antibodies that mimic CD4 binding. Science.

[14] Hoot, S. et al. Recombinant HIV envelope proteins fail to engage germline versions of anti-CD4bs bNAbs. *PLoS Pathog*. 10.1371/journal.ppat.1003106 (2013). 10.1371/journal.ppat.1003106 PMC3536657 23300456

[15] McGuire AT (2013). Engineering HIV envelope protein to activate germline B cell receptors of broadly neutralizing anti-CD4 binding site antibodies. J. Exp. Med..

[16] Jardine J (2013). Rational HIV immunogen design to target specific germline B cell receptors. Science.

[17] Sliepen K (2015). Binding of inferred germline precursors of broadly neutralizing HIV-1 antibodies to native-like envelope trimers. Virology.

[18] Havenar-Daughton C, Lee JH, Crotty S (2017). Tfh cells and HIV bnAbs, an immunodominance model of the HIV neutralizing antibody generation problem. Immunol. Rev..

[19] Burton, D. R. What Are the Most Powerful Immunogen Design Vaccine Strategies? *Cold Spring Harb. Perspect. Biol*. 10.1101/cshperspect.a030262 (2017). 10.1101/cshperspect.a030262 PMC5540812 28159875

[20] Stamatatos L, Pancera M, McGuire AT (2017). Germline-targeting immunogens. Immunol. Rev..

[21] Steichen JM (2016). HIV vaccine design to target germline precursors of glycan-dependent broadly neutralizing antibodies. Immunity.

[22] Steichen JM (2019). A generalized HIV vaccine design strategy for priming of broadly neutralizing antibody responses. Science.

[23] Jardine JG (2016). HIV-1 broadly neutralizing antibody precursor B cells revealed by germline-targeting immunogen. Science.

[24] McGuire AT (2014). Antigen modification regulates competition of broad and narrow neutralizing HIV antibodies. Science.

[25] McGuire, A. T. et al. Specifically modified Env immunogens activate B-cell precursors of broadly neutralizing HIV-1 antibodies in transgenic mice. *Nat. Commun*. 10.1038/ncomms10618 (2016). 10.1038/ncomms10618 PMC4770077 26907590

[26] Medina-Ramírez M (2017). Design and crystal structure of a native-like HIV-1 envelope trimer that engages multiple broadly neutralizing antibody precursors in vivo. J. Exp. Med..

[27] Jardine JG (2015). HIV-1 vaccines. Priming a broadly neutralizing antibody response to HIV-1 using a germline-targeting immunogen. Science.

[28] Watson CT, Glanville J, Marasco WA (2017). The individual and population genetics of antibody immunity. Trends Immunol..

[29] Abbott RK (2018). Precursor frequency and affinity determine B cell competitive fitness in germinal centers, tested with germline-targeting HIV vaccine immunogens. Immunity.

[30] Dosenovic P (2018). Anti-HIV-1 B cell responses are dependent on B cell precursor frequency and antigen-binding affinity. Proc. Natl Acad. Sci. USA.

[31] Havenar-Daughton C, Abbott RK, Schief WR, Crotty S (2018). When designing vaccines, consider the starting material: the human B cell repertoire. Curr. Opin. Immunol..

[32] Sok D (2016). Priming HIV-1 broadly neutralizing antibody precursors in human Ig loci transgenic mice. Science.

[33] Sangesland M (2019). Germline-encoded affinity for cognate antigen enables vaccine amplification of a human broadly neutralizing response against influenza virus. Immunity.

[34] Huang D (2020). B cells expressing authentic naive human VRC01-class BCRs can be primed and recruited to germinal centers in multiple independent mouse models. Proc. Natl Acad. Sci. USA.

[35] Duan H (2018). Glycan masking focuses immune responses to the HIV-1 CD4-binding site and enhances elicitation of VRC01-class precursor antibodies. Immunity.

[36] Havenar-Daughton C (2018). The human naive B cell repertoire contains distinct subclasses for a germline-targeting HIV-1 vaccine immunogen. Sci. Transl. Med..

[37] Wu X (2010). Rational design of envelope identifies broadly neutralizing human monoclonal antibodies to HIV-1. Science.

[38] West AP, Diskin R, Nussenzweig MC, Björkman PJ (2012). Structural basis for germ-line gene usage of a potent class of antibodies targeting the CD4-binding site of HIV-1 gp120. Proc. Natl Acad. Sci. USA.

[39] Zhou T (2015). Structural repertoire of HIV-1-neutralizing antibodies targeting the CD4 supersite in 14 donors. Cell.

[40] Yacoob C (2016). Differences in allelic frequency and CDRH3 region limit the engagement of HIV Env immunogens by putative VRC01 neutralizing antibody precursors. Cell Rep..

[41] Setliff I (2019). High-throughput mapping of B cell receptor sequences to antigen specificity. Cell.

[42] Goldstein, L. D. et al. Massively parallel single-cell B-cell receptor sequencing enables rapid discovery of diverse antigen-reactive antibodies. *Commun. Biol*. 10.1038/s42003-019-0551-y (2019). 10.1038/s42003-019-0551-y PMC6689056 31428692

[43] Dosenovic P (2019). Anti-idiotypic antibodies elicit anti-HIV-1–specific B cell responses. J. Exp. Med..

[44] Cao Y (2020). Potent neutralizing antibodies against SARS-CoV-2 identified by high-throughput single-cell sequencing of convalescent patients’ B cells. Cell.

[45] Stoeckius M (2017). Simultaneous epitope and transcriptome measurement in single cells. Nat. Methods.

[46] Huang J (2016). Identification of a CD4-binding-site antibody to HIV that evolved near-pan neutralization breadth. Immunity.

[47] Georgiev IS (2013). Delineating antibody recognition in polyclonal sera from patterns of HIV-1 isolate neutralization. Science.

[48] Gristick HB (2016). Natively glycosylated HIV-1 Env structure reveals new mode for antibody recognition of the CD4-binding site. Nat. Struct. Mol. Biol..

[49] Sliepen K (2015). Presenting native-like HIV-1 envelope trimers on ferritin nanoparticles improves their immunogenicity. Retrovirology.

[50] Kanekiyo M (2019). Mosaic nanoparticle display of diverse influenza virus hemagglutinins elicits broad B cell responses. Nat. Immunol..

[51] Moyer TJ (2020). Engineered immunogen binding to alum adjuvant enhances humoral immunity. Nat. Med..

[52] Wang Y, Jackson KJL, Sewell WA, Collins AM (2008). Many human immunoglobulin heavy-chain IGHV gene polymorphisms have been reported in error. Immunol. Cell Biol..

[53] Mikocziova I (2021). Polymorphisms in human immunoglobulin heavy chain variable genes and their upstream regions. Nucleic Acids Res..

[54] Schmiedel BJ (2018). Impact of genetic polymorphisms on human immune. Cell Gene Expr. Cell.

[55] Altshuler DL (2010). A map of human genome variation from population-scale sequencing. Nature.

[56] Rodriguez, O. L. et al. A novel framework for characterizing genomic haplotype diversity in the human immunoglobulin heavy chain locus. *Front. Immunol*. 10.3389/fimmu.2020.02136 (2020). 10.3389/fimmu.2020.02136 PMC7539625 33072076

[57] Ohlin, M. Poorly expressed alleles of several human immunoglobulin heavy chain variable (IGHV) genes are common in the human population. *Front. Immunol*. 10.3389/fimmu.2020.603980 (2020). 10.3389/fimmu.2020.603980 PMC7943739 33717051

[58] Sasso EH, Johnson T, Kipps TJ (1997). Expression of an Ig V(H) gene, 51p1, is proportional to its germline gene copy number. Ann. NY Acad. Sci..

[59] Avnir, Y. et al. IGHV1-69 polymorphism modulates anti-influenza antibody repertoires, correlates with IGHV utilization shifts and varies by ethnicity. *Sci. Rep*. 10.1038/srep20842 (2016). 10.1038/srep20842 PMC4754645 26880249

[60] Gidoni, M. et al. Mosaic deletion patterns of the human antibody heavy chain gene locus shown by Bayesian haplotyping. *Nat. Commun*. 10.1038/s41467-019-08489-3 (2019). 10.1038/s41467-019-08489-3 PMC6367474 30733445

[61] Boyd SD (2010). Individual variation in the germline Ig gene repertoire inferred from variable region gene rearrangements. J. Immunol..

[62] Nielsen, S. C. A. et al. Shaping of infant B cell receptor repertoires by environmental factors and infectious disease. *Sci. Transl. Med*. 10.1126/scitranslmed.aat2004 (2019). 10.1126/scitranslmed.aat2004 PMC6733608 30814336

[63] Omer A (2020). VDJbase: an adaptive immune receptor genotype and haplotype database. Nucleic Acids Res..

[64] Kato Y (2020). Multifaceted effects of antigen valency on B cell response composition and differentiation in vivo. Immunity.

[65] Brochet X, Lefranc MP, Giudicelli V (2008). IMGT/V-QUEST: the highly customized and integrated system for IG and TR standardized V-J and V-D-J sequence analysis. Nucleic Acids Res..

[66] Giudicelli, V., Brochet, X. & Lefranc, M. P. IMGT/V-QUEST: IMGT standardized analysis of the immunoglobulin (IG) and T cell receptor (TR) nucleotide sequences. *Cold Spring Harb. Protoc*. 10.1101/pdb.prot5633 (2011). 10.1101/pdb.prot5633 21632778

[67] Trapnell C, Pachter L, Salzberg SL (2009). TopHat: discovering splice junctions with RNA-Seq. Bioinformatics.

[68] Li H (2009). The Sequence Alignment/Map format and SAMtools. Bioinformatics.

[69] Shlemov A (2017). Reconstructing antibody repertoires from error-prone immunosequencing datasets. J. Immunol..

[70] Gadala-Maria, D. et al. Identification of subject-specific immunoglobulin alleles from expressed repertoire sequencing data. *Front. Immunol*. 10.3389/fimmu.2019.00129 (2019). 10.3389/fimmu.2019.00129 PMC6381938 30814994

[71] Crooks GE, Hon G, Chandonia J, Brenner SE (2004). WebLogo: a sequence logo generator. Genome Res..

