# Supplementary material for: Vaccine genetics of IGHV1-2 VRC01-class broadly neutralizing antibody precursor naïve human B cells
Source: NPJ Vaccines. 2021 Sep 6;6:113. doi: 10.1038/s41541-021-00376-7 (PMC8421370; doi:10.1038/s41541-021-00376-7)
Supplement: Supplementary file 1 — Supplementary Information [file 41541_2021_376_MOESM1_ESM.pdf]

SUPPLEMENTARY FIGURES

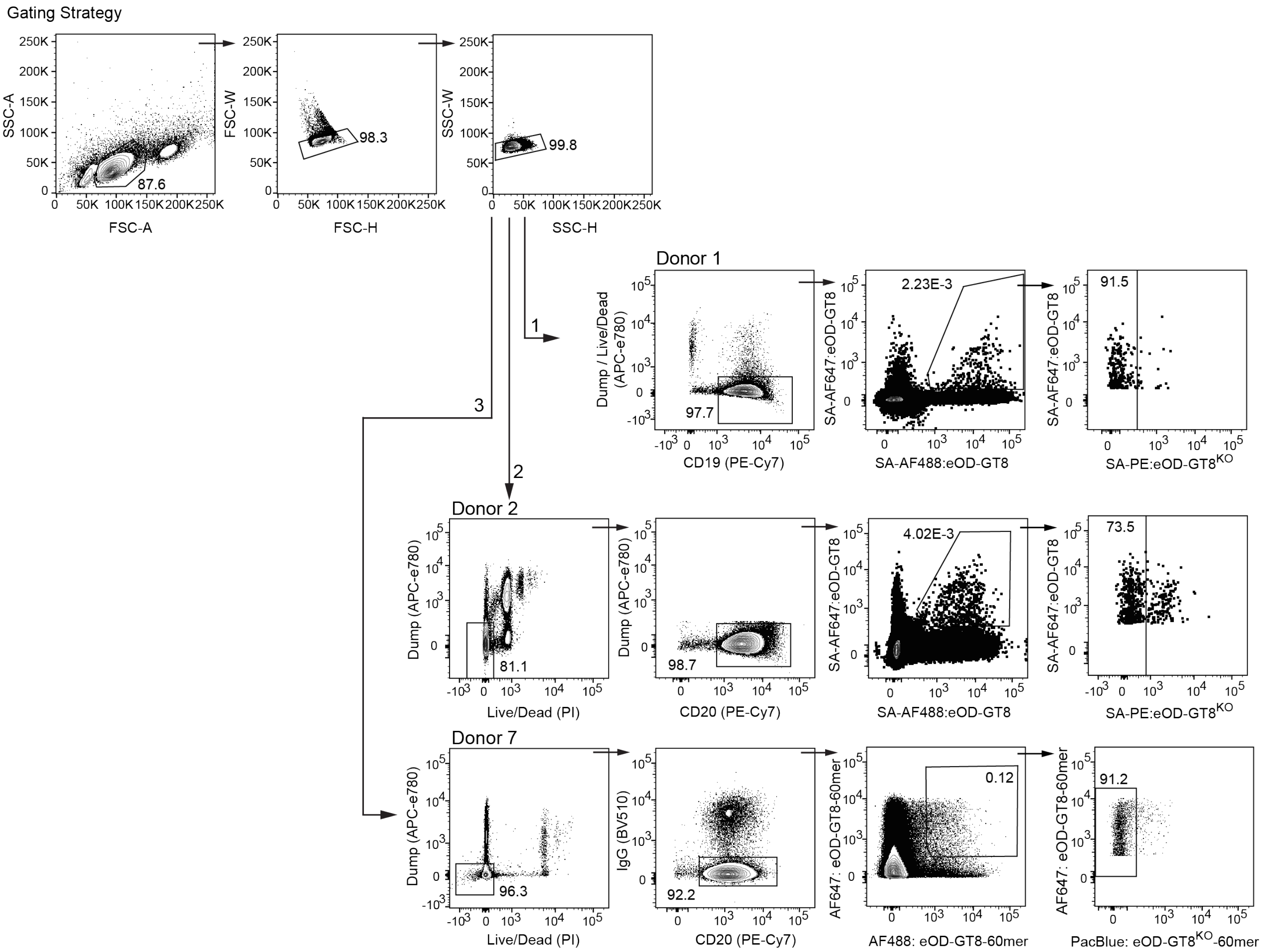

Supplementary Figure 1. Sort gating strategy

Complete gating strategies for sorted samples. Following the common Lymphocyte/Singlet/Singlet gaining (example from donor 1 sample), strategies differed slightly depending on the sample. Samples dated according to strategy 1: donors 1, 4, 8, 9; Samples gated according to strategy 2: donors 2, 3, 5, 6; Samples gated according to strategy 3: donor 7.

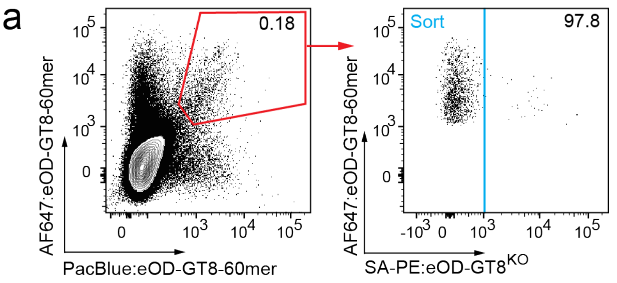

c

LC	LCDR3 Length
IGKV1-39	10
IGKV3-11	7
IGKV3-15	10
IGKV3-15	5
IGKV4-1	9

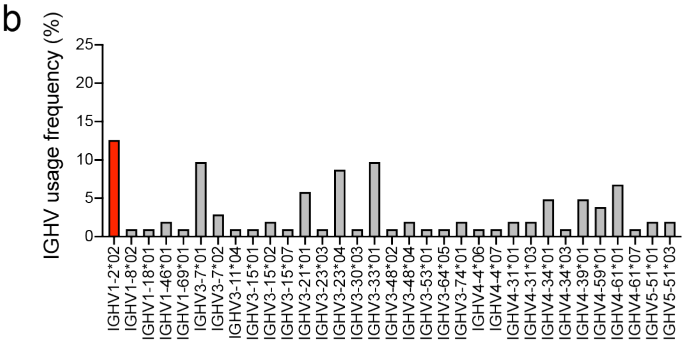

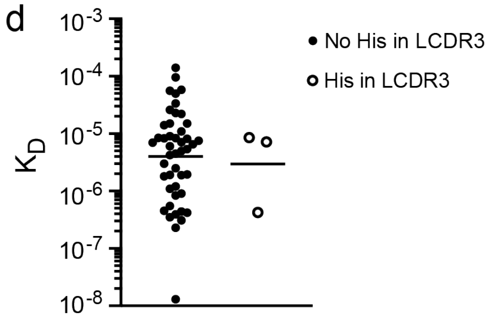

Supplementary Figure 2. Single cell sorting using eOD-GT8-60mer probes.

B cells enriched from healthy donor 8 PBMCs were stained with eOD-GT8-60mer probes directly conjugated with fluorophores and single cell sorted for of IgG^{neg} / eOD-GT8-60mer^{++} eOD-GT8^{KO}-60mer^{neg} B cells.

(a) Flow cytometry of probe-stained B cells. Gated on IgG^{neg} $CD19^{+}$ B cells.

(b) IGHV gene usage distribution among sequenced B cells (n=103).

(c) Successfully recovered LC sequences paired with BCRs expressing IGHV1-2 HC. Only one LC has a short 5-AA LCDR3.

(d) Monomeric dissociation constants for several expressed VRC01-class naïve BCR Fabs binding to eOD-GT8. The BCRs were identified using eOD-GT8 tetramer probes in previous studies[23,36]. Three Abs have an LCDR3 with a histidine residue. Geometric mean is shown.

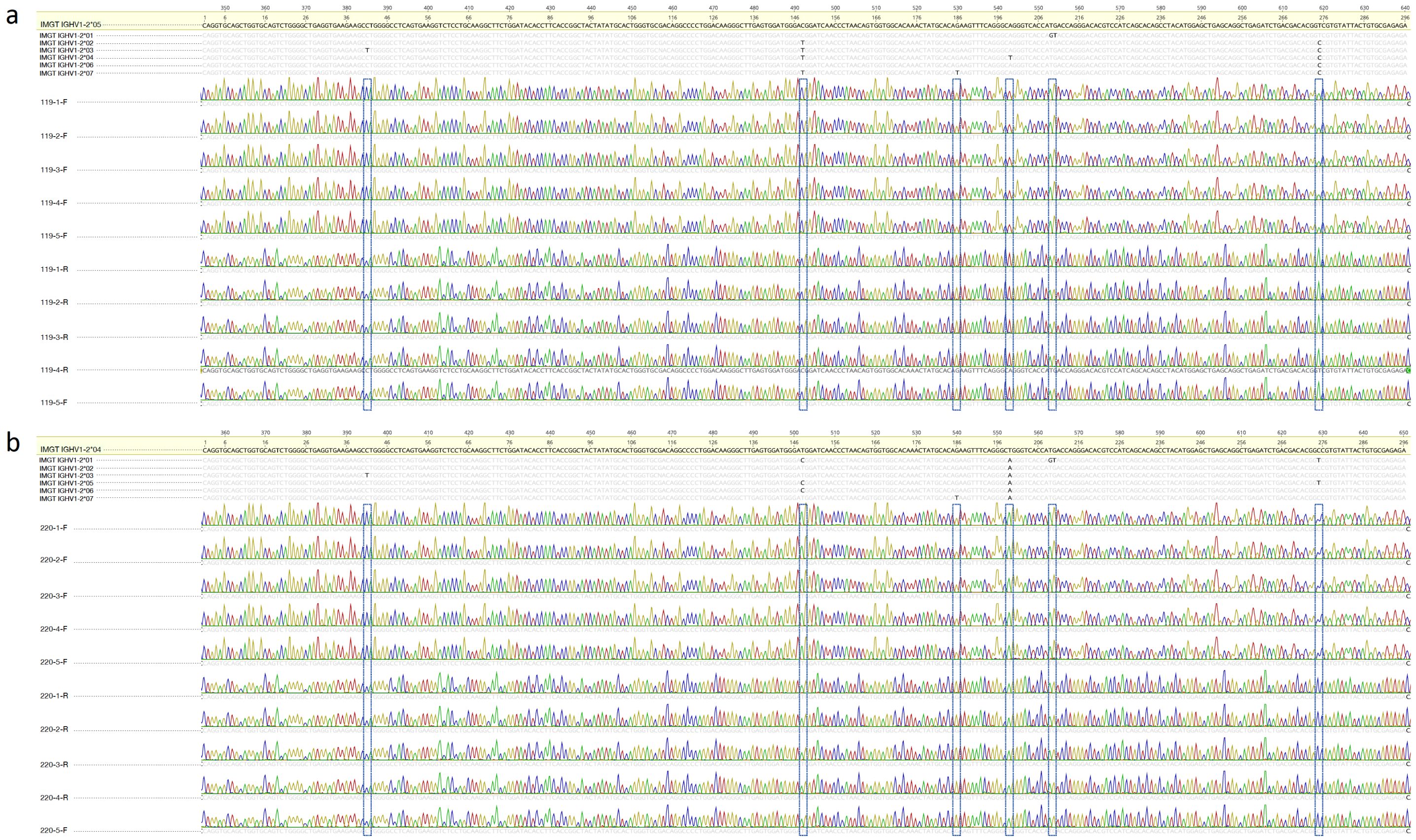

Supplementary Figure 3. IGHV1-2 PCR, cloning, and Sanger sequencing confirm allele genotypes in donors 1 and 9

Sanger chromatograms representing five sequenced clones from targeted IGHV1-2 gene PCR of genomic DNA in donors 9 **(a)** and 1 **(b)**. All clones were sequenced from both the forward and reverse directions to fully span the entirety of the IGHV1-2 coding region. Donor sequences are aligned to all known IGHV1-2 alleles, and SNPs differentiating either *05 or *04 from all other alleles are indicated (dashed boxes). As shown, all sequences within donor 9 **(a)** and donor 1 **(b)** matched *05 and *04, respectively, with 100% sequence identity.

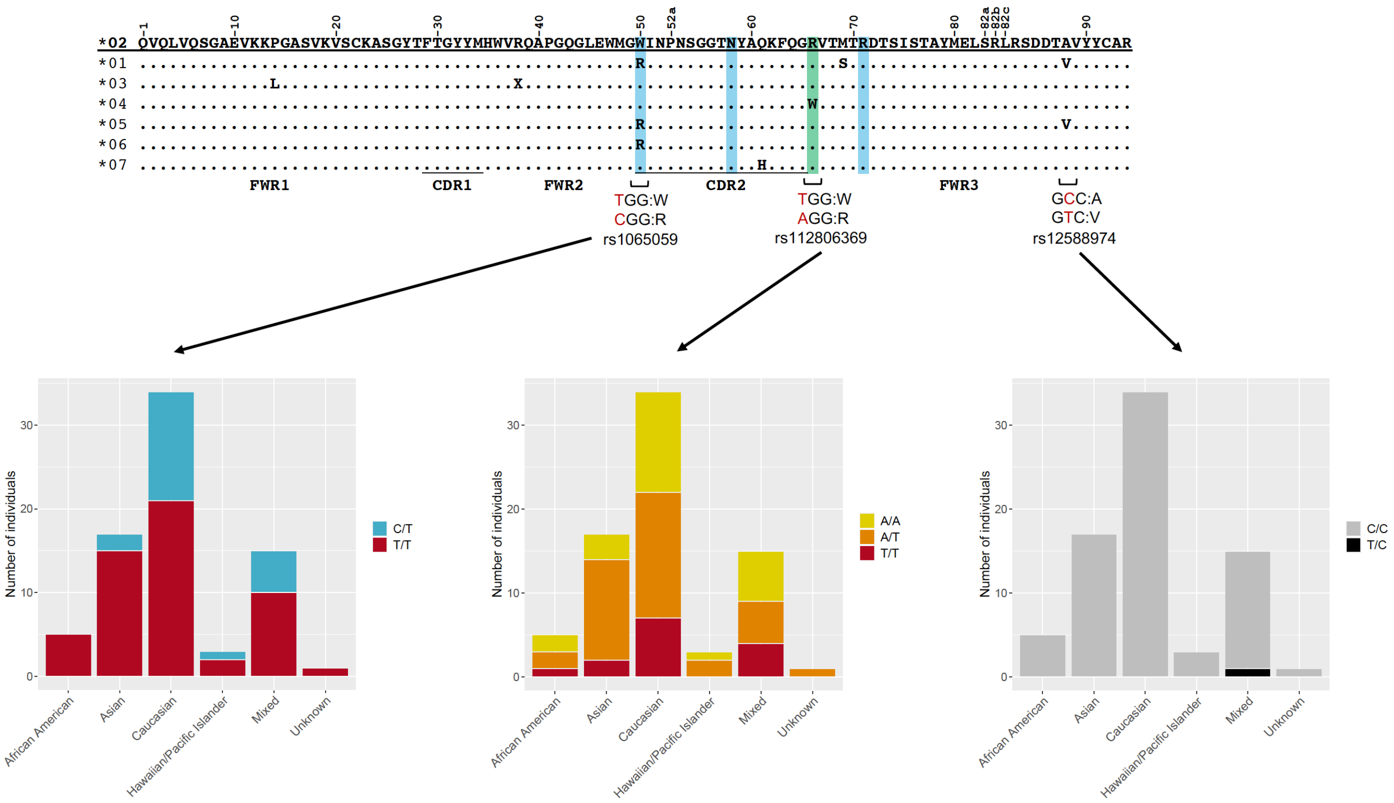

Supplementary Figure 4. IGHV1-2 SNP genotypes inferred from RNA-seq data

AA sequence alignment of the seven known IGHV1-2 alleles, as in Fig. 4a. AA positions represented by SNPs (rs1065059, rs112806369, and rs12588974) present in the RNA-seq data are indicated. Stacked bar plots showing the distribution of inferred SNP genotypes within each population subgroup (n=75; African American, n=5; Asian, n=17; Caucasian, n=34; Hawaiian/Pacific Islander, n=3; Mixed ethnicity, n=15; Unknown, n=1).

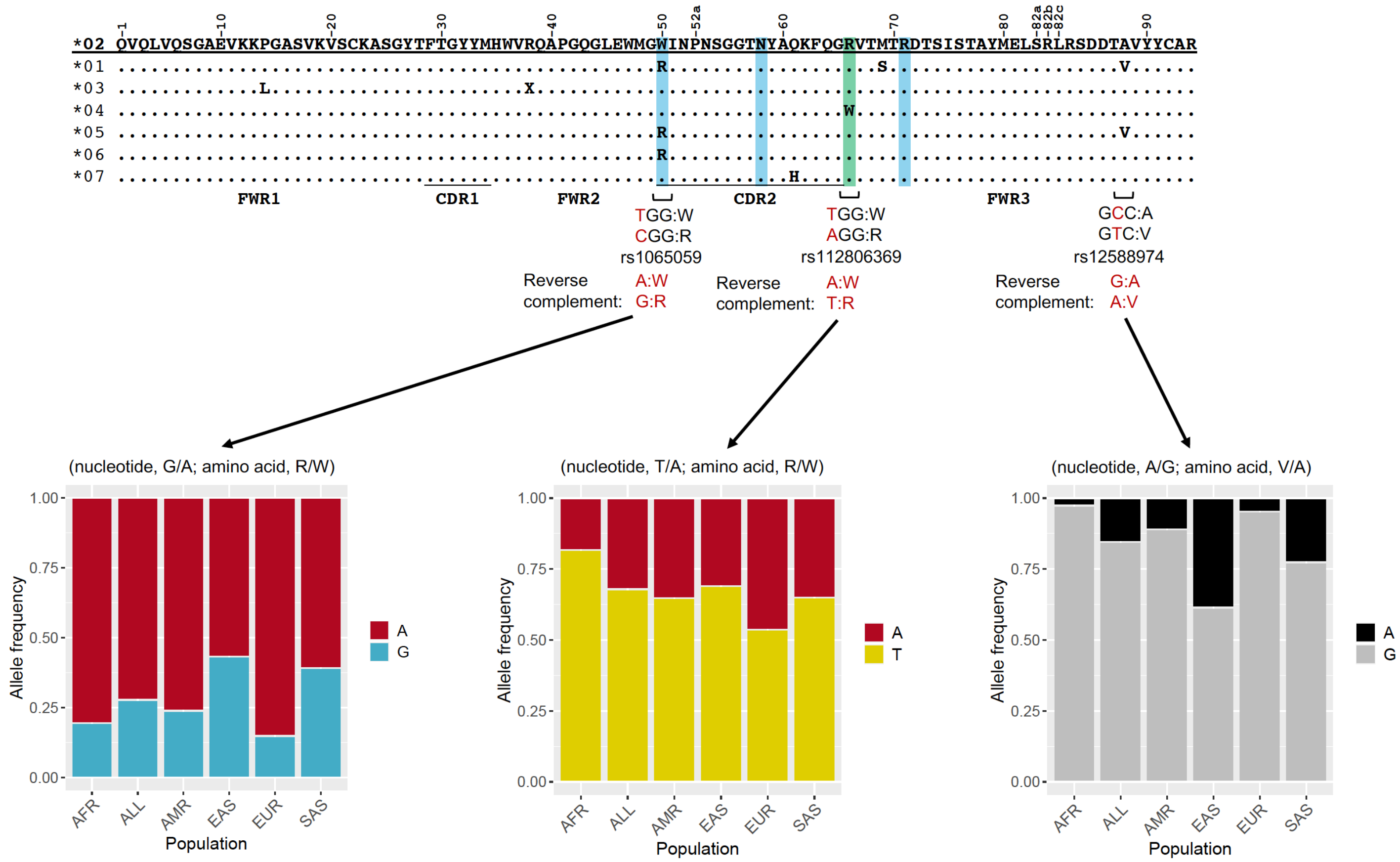

Supplementary Figure 5. IGHV1-2 SNP allele frequency distributions in human subpopulations represented in 1000 Genomes Project data

AA sequence alignment of the seven known IGHV1-2 alleles, as in Fig. 4a. AA positions represented by SNPs rs1065059, rs112806369, and rs12588974 are indicated. Reverse complement bases for each SNP are provided; the 1KGP variant call set contains complement alleles to reflect the fact that IGHV1-2 is found in reverse orientation (3′-5′) in the genome reference assembly. Stacked bar charts displaying the allele frequencies at each SNP within the overall population, as well as five broad human subpopulations are shown (All, n=5,008; AFR, African, n=1,322; AMR, American, n=694; EAS, East Asian, n=1,008; EUR, European, n=1,006; SAS, South Asian, n=978).

SUPPLEMENTARY TABLES

	IGHV	IGHD	IGHJ	CDRH3	IGKV	IGKJ	CDRL3	HC GenBank	LC GenBank
**D1-01**	IGHV1-2*04	IGHD4-17*01	IGHJ2*01	CARADYGDYWYFDLW	IGKV1-5*01	IGKJ1*01	CQQYETF	MZ594686	MZ594787
**D1-02**	IGHV1-2*04	IGHD1-14*01 ORF	IGHJ5*02	CARATRGHFSFDPW	IGKV3-20*01	IGKJ2*01	CQQYETF	MZ594687	MZ594788
**D1-03**	IGHV1-2*04	IGHD6-13*01	IGHJ2*01	CARDRGNSSSWHYWYFDLW	IGKV1D-33*01	IGKJ4*01	CQQYDSF	MZ594688	MZ594789
**D1-04**	IGHV1-2*04	IGHD6-19*01	IGHJ4*02	CARDRRLGSGWYFDYW	IGKV1D-33*01	IGKJ3*01	CQQYKTF	MZ594689	MZ594790
**D1-05**	IGHV1-2*04	IGHD4-17*01	IGHJ3*02	CARDYGDPHAFDIW	IGKV3-20*01	IGKJ2*01	CQQYDTF	MZ594690	MZ594791
**D1-06**	IGHV1-2*04	IGHD3-22*01	IGHJ4*02	CAREDDSSGYFYW	IGKV3-20*01	IGKJ2*01	CQQYQTF	MZ594691	MZ594792
**D1-07**	IGHV1-2*04	IGHD3-10*02	IGHJ3*02	CAREYYYDRGKAGAFDIW	IGKV3-20*01	IGKJ4*01	CQQYDTF	MZ594692	MZ594793
**D1-08**	IGHV1-2*04	IGHD1-26*01	IGHJ4*03	CARGEYSGSYYAIW	IGKV1-5*01	IGKJ1*01	CQQPGTF	MZ594693	MZ594794
**D1-09**	IGHV1-2*04	IGHD2-15*01	IGHJ4*02	CARGYEAAATYDYW	IGKV3-20*01	IGKJ2*01	CQQYGTF	MZ594694	MZ594795
**D1-10**	IGHV1-2*04	IGHD4-17*01	IGHJ4*02	CARRSRDYGANVGYDYW	IGKV3-20*01	IGKJ2*01	CQQYEAF	MZ594695	MZ594796
**D1-11**	IGHV1-2*04	IGHD6-19*01	IGHJ5*02	CARSRHSSASAFDPW	IGKV1D-33*01	IGKJ2*01	CQQYDTF	MZ594696	MZ594797
**D1-12**	IGHV1-2*04	IGHD6-19*01	IGHJ4*02	CARSTEDSSGWYGYW	IGKV1-5*01	IGKJ4*01	CQHYGAF	MZ594697	MZ594798
**D1-13**	IGHV1-2*04	IGHD2-2*01	IGHJ3*02	CARTSRLGDAFDIW	IGKV1-5*01	IGKJ3*01	CQQYETF	MZ594698	MZ594799
**D1-14**	IGHV1-2*04	IGHD2-2*01	IGHJ3*02	CARVCSSTSCYGAFDIW	IGKV3-15*01	IGKJ3*01	CQQYNTF	MZ594699	MZ594800
**D1-15**	IGHV1-2*04	IGHD6-19*01	IGHJ2*01	CARVSIAVAGWYFDLW	IGKV3-20*01	IGKJ1*01	CQQYRTF	MZ594700	MZ594801
**D1-16**	IGHV1-2*04	IGHD3-10*01	IGHJ6*02	CASGGGGGSGDYYYYGMDVW	IGKV3-20*01	IGKJ2*01	CQQYEAF	MZ594701	MZ594802
**D1-17**	IGHV1-2*04	IGHD2-8*01	IGHJ4*02	CASGPKGLDYW	IGKV1D-33*01	IGKJ3*01	CQQFGAF	MZ594702	MZ594803
**D1-18**	IGHV1-2*04	IGHD1-26*01	IGHJ4*02	CARVGSYYDIYYFDYW	IGLV2-8*02	IGLJ2*01	CSYADLF	MZ594703	MZ594804
**D1-19**	IGHV1-2*04	IGHD6-25*01	IGHJ3*02	CARFSSGGGGRNAFDIW	IGLV2-8*02	IGLJ2*01	CSYAALF	MZ594704	MZ594805
**D1-20**	IGHV1-2*04	IGHD3-10*01	IGHJ4*02	CASRGSGSYYETW	IGLV2-14*02	IGLJ1*01	CSSSEVF	MZ594705	MZ594806
**D2-01**	IGHV1-2*02	IGHD5-12*01	IGHJ4*02	CARAVGSFRGYSGWKFDYW	IGKV3-20*01	IGKJ5*01	CQQYGSF	MZ594706	MZ594807
**D2-02**	IGHV1-2*02	IGHD3-10*01	IGHJ5*02	CARAVIEKLWFGELRGYWFDPW	IGKV1-5*01	IGKJ3*01	CQQYETF	MZ594707	MZ594808
**D2-03**	IGHV1-2*02	IGHD5-12*01	IGHJ4*02	CARDKRPLVATIESGLYLDYW	IGKV1-5*01	IGKJ4*01	CQQYNSF	MZ594708	MZ594809
**D2-04**	IGHV1-2*02	IGHD6-13*01	IGHJ4*02	CARDWGVDSSSWPGFDYW	IGKV3-20*01	IGKJ1*01	CQQYEAF	MZ594709	MZ594810
**D2-05**	IGHV1-2*02	IGHD3-10*01	IGHJ4*02	CAREPGPLGEYGYW	IGKV1-9*01	IGKJ5*01	CQQLSTF	MZ594710	MZ594811
**D2-06**	IGHV1-2*02	IGHD3-3*01	IGHJ4*02	CARGGVVIDYW	IGKV3-20*01	IGKJ5*01	CQQYGLF	MZ594711	MZ594812
**D2-07**	IGHV1-2*02	IGHD6-19*01	IGHJ4*02	CARGRGRYFDWLFW	IGKV1D-33*01	IGKJ4*01	CQQYDNF	MZ594712	MZ594813
**D2-08**	IGHV1-2*02	IGHD6-19*01	IGHJ4*02	CARIPLYSSGWYDYW	IGKV1-12*01	IGKJ3*01	CQQANSF	MZ594713	MZ594814
**D2-09**	IGHV1-2*02	IGHD3-16*01	IGHJ2*01	CARLSKGGFGGGANWYFDLW	IGKV1D-33*01	IGKJ4*01	CQQYDSF	MZ594714	MZ594815
**D2-10**	IGHV1-2*02	IGHD3-10*01	IGHJ4*02	CARMQYYGSGSYSGW	IGKV4-1*01	IGKJ3*01	CQQSETF	MZ594715	MZ594816
**D2-11**	IGHV1-2*02	IGHD6-19*01	IGHJ4*02	CARVDSTRSSGWPIDYW	IGKV1-5*01	IGKJ3*01	CQQYNSF	MZ594716	MZ594817
**D2-12**	IGHV1-2*02	IGHD3-22*01	IGHJ4*02	CARVGFYYDSSGYYSYW	IGKV4-1*01	IGKJ2*01	CQQYYTF	MZ594717	MZ594818
**D2-13**	IGHV1-2*02	IGHD5-24*01 ORF	IGHJ2*01	CARVGGDGYKSYWYFDLW	IGKV3-20*01	IGKJ1*01	CQQYGTF	MZ594718	MZ594819
**D2-14**	IGHV1-2*02	IGHD1-26*01	IGHJ3*02	CARWVGATTSDNDAFDIW	IGKV1-5*01	IGKJ1*01	CQQYNAF	MZ594719	MZ594820
**D2-15**	IGHV1-2*02	IGHD4-17*01	IGHJ4*02	CARYDYGDYWAFDYW	IGKV1-5*01	IGKJ4*01	CQQYRTF	MZ594720	MZ594821
**D2-16**	IGHV1-2*02	IGHD6-13*01	IGHJ3*02	CAWQQLVNAFDIW	IGKV1D-33*01	IGKJ3*01	CQQYDFF	MZ594721	MZ594822
**D2-17**	IGHV1-2*04	IGHD3-22*01	IGHJ3*02	CARAGNYDSSGYYRPAFDIW	IGKV1-39*01	IGKJ4*01	CQQSYTF	MZ594722	MZ594823
**D2-18**	IGHV1-2*04	IGHD3-3*01	IGHJ4*02	CARASNYDFWSGYTYW	IGKV1-5*01	IGKJ3*01	CQHPETF	MZ594723	MZ594824
**D2-19**	IGHV1-2*04	IGHD4-17*01	IGHJ2*01	CARGDYGDYLGHSDHAWYFDLW	IGKV3-20*01	IGKJ2*03	CQQYEGF	MZ594724	MZ594825
**D2-20**	IGHV1-2*04	IGHD1-26*01	IGHJ4*02	CARGSGSYYDYW	IGKV1-9*01	IGKJ4*01	CQQLNSF	MZ594725	MZ594826
**D3-01**	IGHV1-2*02	IGHD4-23*01 ORF	IGHJ4*02	CARFYGGNSCFDYW	IGKV1-12*01	IGKJ4*01	CQQANSF	MZ594726	MZ594827
**D3-02**	IGHV1-2*02	IGHD3-22*01	IGHJ3*02	CAGNVYYYDSSGYYFWAFDIW	IGKV1-5*01	IGKJ2*03	CQQYNSF	MZ594727	MZ594828
**D3-03**	IGHV1-2*02	IGHD3-10*01	IGHJ5*02	CARARTGGSGSYVFDPW	IGKV1-5*01	IGKJ2*01	CQQYEFF	MZ594728	MZ594829
**D3-04**	IGHV1-2*02	IGHD1-26*01	IGHJ3*02	CARGGSHGAFDIW	IGKV1-5*01	IGKJ3*01	CQQSSTF	MZ594729	MZ594830
**D3-05**	IGHV1-2*02	IGHD1-1*01	IGHJ5*02	CASNHRTYKQQLELSRWEFDPW	IGKV1-5*01	IGKJ2*02	CQQYGTF	MZ594730	MZ594831
**D3-06**	IGHV1-2*02	IGHD6-6*01	IGHJ2*01	CARDRGGQLVGWYFDLW	IGKV1D-33*01	IGKJ2*01	CQQYFSF	MZ594731	MZ594832
**D3-07**	IGHV1-2*02	IGHD2-15*01	IGHJ4*02	CARVGAAASWYFDYW	IGKV1D-33*01	IGKJ4*01	CQQYDNF	MZ594732	MZ594833
**D3-08**	IGHV1-2*02	IGHD6-19*01	IGHJ4*02	CARYEQWLVWYYFDYW	IGKV3-15*01	IGKJ2*01	CQQYNNF	MZ594733	MZ594834
**D3-09**	IGHV1-2*02	IGHD3-22*01	IGHJ3*02	CARAPYDSSGYYYWAFDIW	IGKV3-20*01	IGKJ2*01	CQQFSFF	MZ594734	MZ594835
**D3-10**	IGHV1-2*02	IGHD3-22*01	IGHJ4*02	CARVLGNYYDSSGYYYGYW	IGKV3-20*01	IGKJ1*01	CQQFETF	MZ594735	MZ594836
**D3-11**	IGHV1-2*02	IGHD1-26*01	IGHJ2*01	CARVLPLNGIVGATTGWYFDLW	IGKV3-20*01	IGKJ2*02	CQSLGTF	MZ594736	MZ594837
**D3-12**	IGHV1-2*02	IGHD6-13*01	IGHJ5*02	CARVTAAALFDPW	IGKV3-20*01	IGKJ2*01	CQHPYTF	MZ594737	MZ594838
**D3-13**	IGHV1-2*02	IGHD1-26*01	IGHJ4*02	CASRSNIVGAALGYW	IGKV3-20*01	IGKJ4*01	CQQYGSF	MZ594738	MZ594839
**D3-14**	IGHV1-2*02	IGHD6-19*01	IGHJ4*02	CAREKGYSSGWSFDYW	IGKV4-1*01	IGKJ4*01	CQQYGTF	MZ594739	MZ594840
**D3-15**	IGHV1-2*02	IGHD6-6*01	IGHJ4*02	CASTQLAAWSFDYW	IGKV4-1*01	IGKJ2*01	CQLGNTF	MZ594740	MZ594841
**D3-16**	IGHV1-2*04	IGHD2-15*01	IGHJ4*02	CARARDCSGGSCYEFSYW	IGKV1-5*01	IGKJ1*01	CQQYEAF	MZ594741	MZ594842
**D3-17**	IGHV1-2*04	IGHD6-19*01	IGHJ4*02	CARYIAVAGTFDYW	IGKV1-5*01	IGKJ2*01	CQQFYTF	MZ594742	MZ594843
**D3-18**	IGHV1-2*04	IGHD1-26*01	IGHJ2*01	CARGGGGSYFTWYFDLW	IGKV3-20*01	IGKJ4*01	CQQYSTF	MZ594743	MZ594844
**D3-19**	IGHV1-2*04	IGHD3-10*01	IGHJ4*02	CARGSTLWFGELLGKYFDYW	IGKV3-20*01	IGKJ3*01	CQQYDTF	MZ594744	MZ594845
**D3-20**	IGHV1-2*02	IGHD6-6*01	IGHJ4*02	CARDIIASSSSSESFWSFDYW	IGLV4-60*02	IGLJ2*01	CQTWALF	MZ594745	MZ594846
**D3-21**	IGHV1-2*02	IGHD5-12*01	IGHJ4*02	CARGDSGYDFDYW	IGLV2-14*02	IGLJ3*02	CSSYKVF	MZ594746	MZ594847
**D3-22**	IGHV1-2*04	IGHD4-23*01 ORF	IGHJ4*02	CARDNPPFDFDYW	IGLV2-23*01	IGLJ2*01	CCSYVVF	MZ594747	MZ594848

Supplementary Table 1. VRC01-class naïve precursor B cell sequences identified using tetramers

	IGHV	IGHD	IGHJ	CDRH3	IGKV	IGKJ	CDRL3	HC GenBank	LC GenBank
**D4-01**	IGHV1-2*02	IGHD3-22*01	IGHJ4*02	CARAGRYYDSSGYYQPFDYW	IGKV3-20*01	IGKJ3*01	CQQYGSF	MZ594748	MZ594849
**D4-02**	IGHV1-2*02	IGHD3-16*01	IGHJ4*02	CARALFGGATGLGPYYFDYW	IGKV1-5*01	IGKJ1*01	CQQYETF	MZ594749	MZ594850
**D4-03**	IGHV1-2*02	IGHD3-10*01	IGHJ5*02	CARDFRDGDTMVRGVIWTFDPW	IGKV1-9*01	IGKJ2*01	CQQLETF	MZ594750	MZ594851
**D4-04**	IGHV1-2*02	IGHD6-13*01	IGHJ4*02	CARDLGIAAGFDYW	IGKV3-20*01	IGKJ5*01	CQQYNTF	MZ594751	MZ594852
**D4-05**	IGHV1-2*02	IGHD3-10*01	IGHJ2*01	CARDPNVESDWYFDLW	IGKV1-5*01	IGKJ1*01	CQQYTTF	MZ594752	MZ594853
**D4-06**	IGHV1-2*02	IGHD3-10*01	IGHJ1*01	CAREDGSGTEYFQHW	IGKV1-5*01	IGKJ2*02	CQQYSTF	MZ594753	MZ594854
**D4-07**	IGHV1-2*02	IGHD4-17*01	IGHJ5*02	CAREVRDDYGANGGWFDPW	IGKV1-5*01	IGKJ4*01	CQHPGTF	MZ594754	MZ594855
**D4-08**	IGHV1-2*04	IGHD5-12*01	IGHJ6*02	CARGGYSGYDENYYYYYGMDVW	IGKV1-5*01	IGKJ1*01	CQQYETF	MZ594755	MZ594856
**D4-09**	IGHV1-2*02	IGHD3-10*02	IGHJ5*02	CARGRLRSGGANGFDPW	IGKV3-20*01	IGKJ1*01	CQQYETF	MZ594756	MZ594857
**D4-10**	IGHV1-2*02	IGHD1-26*01	IGHJ4*02	CARLYSGSYYGGYYFDYW	IGKV3-20*01	IGKJ2*01	CQQYALF	MZ594757	MZ594858
**D4-11**	IGHV1-2*02	IGHD3-22*01	IGHJ4*02	CARPAYYYDSSGYYDYW	IGKV1-5*01	IGKJ1*01	CQQSGAF	MZ594758	MZ594859
**D4-12**	IGHV1-2*02	IGHD6-6*01	IGHJ4*02	CARPHIAARYYFDYW	IGKV4-1*01	IGKJ2*01	CQQEYTF	MZ594759	MZ594860
**D4-13**	IGHV1-2*02	IGHD3-16*01	IGHJ5*02	CARPLTFGGVMMRNWFDPW	IGKV1-5*01	IGKJ1*01	CQQYTTF	MZ594760	MZ594861
**D4-14**	IGHV1-2*02	IGHD3-9*01	IGHJ3*02	CARPQDWSRAFDIW	IGKV1-5*01	IGKJ1*01	CQQSWTF	MZ594761	MZ594862
**D4-15**	IGHV1-2*02	IGHD5-18*01	IGHJ6*02	CARRGYTLHYYYYYGMDVW	IGKV3-11*01	IGKJ3*01	CQQADTF	MZ594762	MZ594863
**D4-16**	IGHV1-2*02	IGHD2-2*01	IGHJ6*02	CARVSSLPYYYYYGMDVW	IGKV3-20*01	IGKJ1*01	CQQYGTF	MZ594763	MZ594864
**D4-17**	IGHV1-2*02	IGHD3-22*01	IGHJ4*02	CARYWAARPDDSSGYYYIDYW	IGKV1-27*01	IGKJ1*01	CQNPGTF	MZ594764	MZ594865
**D4-18**	IGHV1-2*02	IGHD5-24*01ORF	IGHJ5*02	CATTDGYNYWWFDPW	IGKV3-20*01	IGKJ2*01	CQQWDTF	MZ594765	MZ594866
**D4-19**	IGHV1-2*02	IGHD1-7*01	IGHJ6*03	CAVSAKLELRRYYYYYMDVW	IGKV3-15*01	IGKJ2*01	CQQYETF	MZ594766	MZ594867
**D4-20**	IGHV1-2*02	IGHD1-26*01	IGHJ4*02	CARDPVGATTHFDYW	IGLV3-1*01	IGLJ2*01	CQAWEVF	MZ594767	MZ594868
**D5-01**	IGHV1-2*02	IGHD6-13*01	IGHJ6*02	CARAVAGSSWYRDYYYYGMDVW	IGKV1-5*01	IGKJ1*01	CQHHGAF	MZ594768	MZ594869
**D5-02**	IGHV1-2*02	IGHD4-17*01	IGHJ3*02	CARDSTAYDAFDIW	IGKV1-5*01	IGKJ1*01	CQHLETF	MZ594769	MZ594870
**D5-03**	IGHV1-2*02	IGHD3-22*01	IGHJ3*02	CARGVPYYDSSGYYWSIW	IGKV3-15*01	IGKJ1*01	CQHQGTF	MZ594770	MZ594871
**D5-04**	IGHV1-2*02	IGHD6-19*01	IGHJ4*02	CARESFLIAVAGTTFDYW	IGKV1-5*01	IGKJ2*01	CQHQGTF	MZ594771	MZ594872
**D5-05**	IGHV1-2*02	IGHD3-10*01	IGHJ6*02	CARDRIITMVRGVISHYYYGMDVW	IGKV1-5*01	IGKJ1*01	CQQSGTF	MZ594772	MZ594873
**D5-06**	IGHV1-2*02	IGHD6-19*01	IGHJ4*02	CARDFSGWYGYW	IGKV3-20*01	IGKJ1*01	CQQSGTF	MZ594773	MZ594874
**D5-07**	IGHV1-2*02	IGHD6-19*01	IGHJ5*02	CARDQEGYSSGWYGTW	IGKV3-20*01	IGKJ1*01	CQQYGAF	MZ594774	MZ594875
**D6-01**	IGHV1-2*02	IGHD5-12*01	IGHJ2*01	CARPTRRGYDYDSWWYFDLW	IGKV1-39*01	IGKJ3*01	CQQSWTF	MZ594775	MZ594876
**D6-02**	IGHV1-2*02	IGHD3-22*01	IGHJ4*02	CARDPPYDSSGYYRWGFDYW	IGKV3-20*01	IGKJ1*01	CQQYGTF	MZ594776	MZ594877
**D6-03**	IGHV1-2*04	IGHD2-15*01	IGHJ3*02	CARWTRNCSGGSCYLYRSGAFDIW	IGKV1-5*01	IGKJ2*01	CQQHHTF	MZ594777	MZ594878
**D6-04**	IGHV1-2*04	IGHD4-17*01	IGHJ2*01	CARTTGSSYWYFDLW	IGKV3-20*01	IGKJ1*01	CQQYASF	MZ594778	MZ594879
**D6-05**	IGHV1-2*02	IGHD4-11*01	IGHJ2*01	CARPIHDYSNYNPDWYFDLW	IGLV3-1*01	IGLJ1*01	CQARNVF	MZ594779	MZ594880
**D7-01**	IGHV1-2*02	IGHD6-13*01	IGHJ4*02	CARDFPASYSSSWYYDYW	IGKV1D-33*01	IGKJ3*01	CQQYDNF	MZ594780	MZ594881
**D7-02**	IGHV1-2*02	IGHD6-13*01	IGHJ4*01	CARDRGHSSSTSFDYW	IGKV1D-33*01	IGKJ2*01	CQQYEAF	MZ594781	MZ594882
**D7-03**	IGHV1-2*02	IGHD6-6*01	IGHJ5*02	CARSPYSSSFDPW	IGKV3-20*01	IGKJ2*03	CQQSDSF	MZ594782	MZ594883
**D7-04**	IGHV1-2*02	IGHD6-19*01	IGHJ4*02	CARTDPSGWYDYW	IGKV3-15*01	IGKJ5*01	CQQFESF	MZ594783	MZ594884
**D7-05**	IGHV1-2*02	IGHD4-17*01	IGHJ4*02	CARVTTDYGDYFPW	IGKV3-20*01	IGKJ3*01	CQQYETF	MZ594784	MZ594885
**D7-06**	IGHV1-2*02	IGHD6-6*01	IGHJ4*02	CASSWTSSSPDNYW	IGKV3-20*01	IGKJ4*01	CQQSTVF	MZ594785	MZ594886
**D7-07**	IGHV1-2*02	IGHD3-22*01	IGHJ4*02	CASSYDSSGYHFDYW	IGKV1D-33*01	IGKJ4*01	CQQYGYF	MZ594786	MZ594887

Supplementary Table 2. VRC01-class naïve precursor B cell sequences identified using 60mers

Cohort/Subgroup	N	IGHV1-2*02	IGHV1-2*04	IGHV1-2*05	IGHV1-2*06
**African American[1]**	5	0.6	0.4	0	0
**Asian[1]**	17	0.47058824	0.47058824	0	0.05882353
**Caucasian[1]**	34	0.38235294	0.42647059	0	0.19117647
**Hawaiian/Pacific Islander[1]**	3	0.5	0.33333333	0	0.16666667
**Mixed ethnicity[1]**	15	0.4	0.43333333	0.03333333	0.13333333
**Unknown[1]**	1	0.5	0.5	0	0
**ALL (RNAseq)[1]**	75	0.42666667	0.43333333	0.00666667	0.13333333
**ALL (RepSeq)**	84	0.4047619	0.4702381	0.00595238	0.11904762

Supplementary Table 3. Allele frequency of IGHV1-2 alleles inferred from RNAseq and RepSeq data
[1]Subgroups from RNAseq data.
