# Supplementary material for: Vaccine genetics of IGHV1-2 VRC01-class broadly neutralizing antibody precursor naïve human B cells
Source: NPJ Vaccines. 2021 Sep 6;6:113. doi: 10.1038/s41541-021-00376-7 (PMC8421370; doi:10.1038/s41541-021-00376-7)
Supplement: Supplementary file 2 — Reporting Summary [file 41541_2021_376_MOESM2_ESM.pdf]

nature research

Corresponding author(s): Shane Crotty, Corey T. Watson

Last updated by author(s): May 28, 2021

Reporting Summary

Nature Research wishes to improve the reproducibility of the work that we publish. This form provides structure for consistency and transparency in reporting. For further information on Nature Research policies, see our Editorial Policies and the Editorial Policy Checklist.

Statistics

For all statistical analyses, confirm that the following items are present in the figure legend, table legend, main text, or Methods section.

n/a	Confirmed	
☐	☒	The exact sample size (*n*) for each experimental group/condition, given as a discrete number and unit of measurement
☐	☒	A statement on whether measurements were taken from distinct samples or whether the same sample was measured repeatedly
☐	☒	The statistical test(s) used AND whether they are one- or two-sided<br>*Only common tests should be described solely by name; describe more complex techniques in the Methods section.*
☒	☐	A description of all covariates tested
☐	☒	A description of any assumptions or corrections, such as tests of normality and adjustment for multiple comparisons
☐	☒	A full description of the statistical parameters including central tendency (e.g. means) or other basic estimates (e.g. regression coefficient) AND variation (e.g. standard deviation) or associated estimates of uncertainty (e.g. confidence intervals)
☐	☒	For null hypothesis testing, the test statistic (e.g. *F*, *t*, *r*) with confidence intervals, effect sizes, degrees of freedom and *P* value noted<br>*Give P values as exact values whenever suitable.*
☒	☐	For Bayesian analysis, information on the choice of priors and Markov chain Monte Carlo settings
☒	☐	For hierarchical and complex designs, identification of the appropriate level for tests and full reporting of outcomes
☒	☐	Estimates of effect sizes (e.g. Cohen's *d*, Pearson's *r*), indicating how they were calculated

Our web collection on statistics for biologists contains articles on many of the points above.

Software and code

Policy information about availability of computer code

Data collection	RNA-sequencing and expressed antibody repertoire sequencing datasets were obtained either directly from authors of previously published studies or from public repositories. Genotype data from the 1000 Genomes Project samples were obtained from the ensembl web porta.
Data analysis	VDJ contig compilation and annotation were performed by using open software indicated in the manuscript, and has been cited in the manuscript. A Github link is provided to the code used to link feature barcoding and VDJ information. Analysis of RNA-sequencing and repertoire sequencing data was conducted using published open source software, as indicated in the manuscript. Statistical analyses were conducted using existing python and R libraries. Flow cytometry were analyzed using FlowJo 10.

For manuscripts utilizing custom algorithms or software that are central to the research but not yet described in published literature, software must be made available to editors and reviewers. We strongly encourage code deposition in a community repository (e.g. GitHub). See the Nature Research guidelines for submitting code & software for further information.

Data

Policy information about availability of data

All manuscripts must include a data availability statement. This statement should provide the following information, where applicable:

- Accession codes, unique identifiers, or web links for publicly available datasets
- A list of figures that have associated raw data
- A description of any restrictions on data availability

Antibody sequences of interest are provided in Supplementary tables 1 and 2 along with their respective GenBank accession codes.

Field-specific reporting

Please select the one below that is the best fit for your research. If you are not sure, read the appropriate sections before making your selection.

☒ Life sciences ☐ Behavioural & social sciences ☐ Ecological, evolutionary & environmental sciences

For a reference copy of the document with all sections, see nature.com/documents/nr-reporting-summary-flat.pdf

Life sciences study design

All studies must disclose on these points even when the disclosure is negative.

Sample size	Sample size calculation was not performed. The number of donors was selected based on our observations in our previous studies.
Data exclusions	Total raw data was analyzed. Where data filtering was applied for identifying highly specific BCR sequences, we have described our filtering rationale and what filters were applied within the manuscript.
Replication	Many of the sorting and sequencing experiments described in the study were performed on several different days and by two different people. Blood samples were obtained from unique donors.
Randomization	Healthy donor blood samples were obtained from random unique donor from the San Diego Blood Bank. There was no criteria for recruitment.
Blinding	Blood donor information was completely blinded. Blinding was not possible for experiments using different protein probes, as they were used by the researcher to perform sample preparation.

Reporting for specific materials, systems and methods

We require information from authors about some types of materials, experimental systems and methods used in many studies. Here, indicate whether each material, system or method listed is relevant to your study. If you are not sure if a list item applies to your research, read the appropriate section before selecting a response.

Materials & experimental systems

n/a	Involved in the study	
☐	☒	Antibodies
☒	☐	Eukaryotic cell lines
☒	☐	Palaeontology and archaeology
☒	☐	Animals and other organisms
☐	☒	Human research participants
☒	☐	Clinical data
☒	☐	Dual use research of concern

Methods

n/a	Involved in the study	
☒	☐	ChIP-seq
☐	☒	Flow cytometry
☒	☐	MRI-based neuroimaging

Antibodies

Antibodies used	CD3 (UCHT1, eBiosciences cat# 47-0038-42, APC-e780, 1:100), CD14 (M5E2, Biolegend cat# 301820, APC-Cy7, 1:100), CD16 (CB16, eBiosciences cat# 47-0168-42, APC-e780, 1:100), CD19 (HIB19, eBiosciences cat# 25-0199-42, PE-Cy7 1:100), CD20 (2H7, eBiosciences, PE-Cy7, 1:100), IgG (HP6019, Biolegend cat #490314, APC-Cy7, 1:100, product has been discontinued by manufacturer), IgG (G18-145, BD Biosciences, BV510, 1:100)
Validation	All antibodies are commercially available. All antibodies have been previously validated by manufacturer or commonly used in publications.

Human research participants

Policy information about studies involving human research participants

Population characteristics	For VDJ sequencing analysis, blood was obtained from healthy adult blood donors. We are blinded from any other donor characteristics.
Recruitment	A specific volume of healthy donor blood was requested through the San Diego Blood Bank. There was no other specific recruitment criteria.
Ethics oversight	The study protocol was approved by the LJI IRB.

Note that full information on the approval of the study protocol must also be provided in the manuscript.

Flow Cytometry

Plots

Confirm that:

☒ The axis labels state the marker and fluorochrome used (e.g. CD4-FITC).

☒ The axis scales are clearly visible. Include numbers along axes only for bottom left plot of group (a 'group' is an analysis of identical markers).

☒ All plots are contour plots with outliers or pseudocolor plots.

☒ A numerical value for number of cells or percentage (with statistics) is provided.

Methodology

Sample preparation	Buffy coats were obtained from the San Diego Blood Bank, and blood processing was outsourced to the La Jolla Institute (LJI) blood processing core, where PBMCs were isolated and frozen down. Frozen PBMCs were thawed and washed in R10 to remove residual DMSO. After counting the recovered PBMCs, cells were subjected to a CD19-positive selection using Miltenyi CD19 MicroBeads according to the manufacturer's protocols. Enriched CD19+ cells were resuspended in R10 and stained for FACS according to the protocol stated in the manuscript.
Instrument	BD FACSAria II
Software	FACSDiva, FlowJo 10
Cell population abundance	Abundance of relevant cell populations is described in Tables 2 and 3 in the manuscript.
Gating strategy	Lymphocytes were gated on FSC-A/SSC-A, followed by singlet gating (FSC-W/FSC-H and SSC-W/SSC-H), Dump- (CD14, CD3, CD16, IgG-) live cells (live/dead-), IgG- B cells (CD20 or CD19+), eOD-GT8 probe+ on two distinct fluorophors, followed by an epitope knockout probe negative population.<br>The common gating strategy to all samples is shown in Supplementary Fig 1. Probe-specific gating strategy pertaining to each sample are shown as independent display items throughout the manuscript.

☒ Tick this box to confirm that a figure exemplifying the gating strategy is provided in the Supplementary Information.
